# The effects of plant density and duration of vegetative growth phase on agronomic traits of medicinal cannabis (*Cannabis sativa* L.): A regression analysis

**DOI:** 10.1371/journal.pone.0315951

**Published:** 2024-12-30

**Authors:** Torsten Schober, Achim Präger, Jens Hartung, Simone Graeff-Hönninger

**Affiliations:** 1 Agronomy, Institute of Crop Science, University of Hohenheim, Stuttgart, Germany; 2 Biostatistics, Institute of Crop Science, University of Hohenheim, Stuttgart, Germany; Universitas Sebelas Maret, INDONESIA

## Abstract

Empirical data on the effect of plant density (PD) and length of the vegetative phase (DVP) on plant growth, yield, and cannabinoid concentration of medicinal cannabis (*Cannabis sativa* L.) are still scarce, leading to a lack of specific cultivation recommendations. We conducted two greenhouse experiments to investigate the effect of PD in the range of 12–36 plants m^-2^ (D-trial) and DVP in the range of 1–4 weeks (V-trial) on plant morphology, biomass growth of individual plant organs, and CBD concentration of individual inflorescence fractions. Empirical models for the relationships between the investigated plant traits and PD/DVP were created using linear regression analysis preceded by a lack-of-fit test. An increase in PD led to a linear decrease in inflorescence yield per plant (*p* = 0.02), whereas a positive linear relationship was found for inflorescence yield (*p* = 0.0001) and CBD yield (*p* = 0.0002) per m^2^. Total area yields in the D-trial ranged from 119 to 247 g m^-2^ from lowest to highest PD. DVP showed a positive linear relationship with inflorescence yield on an individual plant (*p* = 0.0001) and area basis (*p* < 0.0001) along with most other relevant agronomic traits such as CBD production, plant size and lateral shoot length. Total area yields in the V-trial ranged from 295 to 571 g m^-2^ from lowest to highest DVP. The yield increase could be linked to the increased inflorescence number per plant rather than inflorescence size. In contrast to expectations, neither PD nor DVP had significant effects on the cannabinoid concentration gradient from upper to lower canopy layers. CBD concentrations in inflorescences from lower canopy layers were reduced by 23% in the V-trial and 46% in the D-trial. However, with increasing PD, the proportion of higher-concentrated inflorescence fractions from upper canopy layers increased from 46% to 68%, while an extension of DVP shifted this proportion only marginally from 45% to 50%. In the context of standardized production, we therefore advocate high-density production systems that increase the proportion of desired inflorescence fractions from upper canopy layers.

## 1. Introduction

*Cannabis sativa* L. (cannabis) is a multifunctional crop whose harvested products are used in various industries (e.g. food, pharmaceuticals, textiles, cosmetics, and others). Its potential use for medicinal purposes has attracted particular interest in recent research endeavors and is based on various groups of secondary metabolites, including cannabinoids, terpenes, flavonoids, and others. Especially cannabinoids have been studied for their therapeutic effects, above all, the most prominent and most frequently encountered representatives Δ9-tetrahydrocannabinol (THC) and cannabidiol (CBD) [1]. In the plant, these chemical compounds are present in their acid forms (THCA, CBDA) and are found in high concentrations primarily in the glandular trichomes of female inflorescences [2]. However, they are also present in vegetative plant organs, albeit to a much lesser extent [2–4]. The production goal can typically be divided into i) inflorescence biomass for the extraction of cannabinoids (and preparation of pharmaceutical formulations), in which case the cannabinoid yield must be maximized, and ii) inflorescences for direct consumption, in which case the standardized composition of the chemical constituents is crucial. Indoor cultivation allows growers to control key environmental factors (e.g. light, temperature, nutrient- and water supply) to steer plant growth toward the desired result and ensure batch-to-batch homogeneity [5]. As most commercially used genotypes are short-day plants, another advantage of indoor production is that the transition from vegetative to generative growth can be controlled at will by lowering the day length (typically from 18 h to 12 h) using artificial lighting concepts.

Light utilization is of central importance for cannabis cultivation. It is a well-known principle that biomass accumulation is directly proportional to the amount of intercepted light. Thus, it is crucial to maximize light interception through optimal leaf coverage of the cultivation area to achieve maximum yields [6]. At the same time, it is known, that a concentration gradient of cannabinoids occurs within the plant, particularly in indoor cultivation under artificial lighting [7–9]. This follows the natural light gradient from the plant’s tip to the stem’s base. Suitable canopy management is therefore required to find a balance between maximizing light interception and light penetration into the lower canopy layers. Another consideration for canopy management strategies concerns the microclimatic conditions (e.g. air movement, temperature, humidity) within the canopy, as these affect the risk of fungal infestation [6, 10] and cannabinoid concentrations [11]. Besides the choice of the cultivated genotype and associated plant morphology, growers can apply several pruning and training techniques or hormone treatments to alter the canopy´s shape [4, 8, 12]. However, in this context, the two most basic factors that growers need to decide on when planning their cultivation system are the choice of planting density (PD) and the total length of the long-day period (vegetative growth).

PD generally influences both the biomass and morphology of individual plants. Morphological changes due to alterations in PD are closely linked to shade avoidance reactions of plants as a response to the reduction in the red:far-red (R:FR) ratio in the deeper layers of the stand [13, 14]. The R:FR ratio is known to be a major factor in triggering shade avoidance symptoms, such as increased internode elongation, petiole elongation, and branching [6, 13, 15]. The impact of PD and R:FR ratio on plant height has been previously demonstrated and discussed for cannabis [7, 16–18]. An increase in PD is accompanied by higher light interception but less light penetration into the lower canopy layers, reducing the photosynthetic activity of the leaves in these layers [6, 10]. As a result, the total biomass of individual plants typically decreases with increasing PD. However, in terms of area, this is compensated for by the higher number of plants, so in general, a positive correlation between PD and the biomass yield per unit area exists [14]. Only at very high PD, a saturation point is reached at which the biomass yield per area remains constant, which is described as the ‘law of constant final yield’ [19]. However, it has been found that in most scientific publications analyzing the effect of PD on plant growth, this saturation point was not reached [14]. For industrial hemp and various production purposes under field conditions, multiple studies have shown that dry inflorescence yield and cannabinoid yield on an area basis continuously increased with PD, while these traits decreased on a per-plant basis [17, 20–23]. So far, not many studies are available that compare the effect of PD on medicinal cannabis in indoor cultivation systems. [7] compared the growth and yield of drug-type cannabis in pot culture for a PD of 1 plant m^-2^ and 2 plants m^-2^. They confirmed the relationship for dry inflorescence yield, as opposed to the cannabinoid yield per m^2^, which was not affected by the density treatments. The authors further showed that the intra-plant variation of cannabinoid concentration of individual inflorescences increased with PD. This was mainly due to a reduced concentration of inflorescences in lower canopy layers, while the inflorescences from the upper layers showed no differences [7]. It is assumed, however, that most indoor growers cultivate their plants at a PD of around or above 15 plants m^-2^ [6, 24]. Guided by these values, [25] compared different genotypes grown under densities of 12 and 16 plants m^-2^ and found that dry area inflorescence yield was not significantly affected by PD, although yield per plant was lower in the higher PD. The same results were found in a later study, where PDs of 16 and 20 plants m^-2^ were compared [26]. To our knowledge, the only analysis of a wider range of PD for indoor cannabis cultivation consists of a meta-analysis of PD between 10 and 20 plants m^-2^ used in scientific publications. The meta-analysis concluded, that PD was not a suitable predictor of final area yield [27]. In general, there is still a lack of empirical data that can map the inflorescence and cannabinoid yield over a wide range of PD being relevant to the production industry and from which quantifiable relationships can be established. Furthermore, the role of PD on the uniformity of inflorescence quality needs to be further elucidated.

The length of the long-day period defines the duration of the vegetative growth phase (DVP) and, consequently, the plant size. It is known that the DVP increases the plant height, width, and number of nodes on the main stem and side shoots as well as the final inflorescence yield per plant. Based on the growth curves of [28], it can be seen that cannabis plants enter a linear growth phase after about 1–2 weeks under long-day conditions for height, lateral shoot, and biomass growth. In addition to the genotype, the selected PD is a fundamental consideration when selecting the DVP. As a rule of thumb, [6] suggested a density of 15 plants m^-2^ and recommended a DVP of 10–15 days if the density is higher and a DVP of 15–30 days if the selected density is lower. However, the effects of DVP on inflorescence area yield or cannabinoid production are still insufficiently described. Based on a meta-analysis, [29] attempted to determine the optimal DVP concerning inflorescence yield and cannabinoid concentration and came to the somewhat surprising conclusion that DVP is positively correlated with cannabinoid concentration but negatively correlated with floral biomass [29]. So far, there is a lack of peer-reviewed literature based on controlled experiments that could form the basis for specific recommendations on DVP.

This study aimed to quantify the influence of PD (12–36 plants m^-2^) and DVP (1–4 weeks) on plant morphology, biomass allocation between plant organs and canopy layers as well as dry inflorescence yield and cannabinoid yield for medicinal cannabis. At the same time, the influence on the uniformity of the inflorescences in terms of size and cannabinoid concentration depending on their position in the canopy was investigated. The main hypotheses were: i) DVP shows a linear relationship with plant biomass and yield traits on an area basis, whereas dry inflorescence yield per square meter will saturate for the high PD of the tested range. ii) PD and DVP both increase the intra-plant gradient of CBD concentration and inflorescence size and negatively affect the homogeneity of yield components. Two greenhouse trials were conducted to test the hypothesis separately for the factors of PD and DVP. The main objective was to derive empirical relationships with target plant traits, which can guide producers in their cultivation management to reach their desired production goals.

## 2. Material and methods

### 2.1 Plant material

Two greenhouse trials were conducted at the Phytotechnikum of the University of Hohenheim. One trial was used to compare selected PD (D-trial), and the other was used to compare different DVP and growth substrates (V-trial). The CBD-rich genotype "Kanada" (AI FAME, Wald-Schönengrund, Switzerland) was used in both trials. This genotype is classified as chemovar III (THC/CBD-ratio < 0.05) and has indica and sativa characteristics in equal measure. In previous studies, this genotype reached CBD concentrations in the range of 6–10% in greenhouse cultivation [4, 30]. The terpene profile of this genotype was previously described by [9]. Planting material was obtained via vegetative propagation. Head cuttings of 10 cm length were cut from 3-month-old mother plants, which were grown exclusively under long-day conditions (18 h day length). Leaves of the cuttings were removed so that only one fully developed leaf and the shoot tip remained. The cuttings were then dipped in 1% Rhizopon AA (Hazerswoude-Rijndijk, Netherlands) and placed in rockwool cubes AO 36/40 (Grodan, Roermond, Netherlands), which had previously been soaked overnight in 0.15% fertilizer solution of Plant Aktiv type A (Hauert, Grossaffoltern, Germany). For rooting, the cuttings were placed in a foil tent where the humidity was kept at ~90% with the help of pond nebulizers. For lighting, ceramic halide lamps CHD Agro 400 (DH Licht GmbH, Wülfrath, Germany) were used, which were adjusted so that the incoming photosynthetically active radiation in the tent was ~100 μmol m^-2^ s^-1^. The day length was set to 18 h. The rooting period for both trials was 14 days.

At the beginning of the experiments, the rooted cuttings were planted in 3-liter pots filled with the substrate used for the respective experiment and soaked in water overnight. A peat-perlite mixture was used as the standard substrate in both experiments. The V-trial used a coconut wood fiber mixture as a second substrate. The mixtures were generously provided by Klasmann-Deilmann (Geeste, Germany). A detailed overview of the composition and properties of the substrates used can be found in the supplementary material (S1 Table). Slow-release fertilizer with the following composition (the same for both substrates) was used for fertilization: 0.5 g L^-1^ Top Substra (Compo Expert, Münster, Germany), 0.67 g L^-1^ Osmocote Bloom 2-3M, 1.33 g L^-1^ Osmocote Exact Hi-End 3-4M and 2.33 g L^-1^ Osmocote Exact Hi-End 5-6M (ICL, Tel Aviv, Israel). The composition and concentration were calculated based on the growth curves for biomass growth and nutrient contents from [28]. Before planting, the water-saturated pots were weighed to determine the pot weight at full container capacity. Irrigation during the trials was done using drip irrigation with one dripper per pot and 36 mL min^-1^ flow rate. Interval and volume were adjusted to keep the water content in the substrate between 60–90% of the maximum container capacity. The plant weight was estimated based on the growth curves of [28] and taken into account when measuring the pot weight.

During the vegetative phases, the plants grew under natural light with supplementary lighting from CHD Agro 400 lamps to keep the day length at 18 h. For flower induction, the trials were moved to a darkroom where the day length could be reduced to 12 h. The only light source here was the CHD Agro 400 lamps. Non-destructive measurements were collected weekly during the trial period. Destructive measurements were taken separately for each trial (section 2.2 & 2.3). In both trials, temperature and air humidity were monitored using Tinytag Plus 2 data loggers (Gemini Data Loggers Ltd., Chichester, West Sussex, UK) and a logging interval of 20 min. Incoming photosynthetic active radiation during the vegetative growth phases was recorded using PAR/LE line sensors (SOLEMS S.A., Palaiseau, France) with a logging interval of 5 min.

### 2.2 Effect of altered planting density (D-trial)

**Fig 1:**
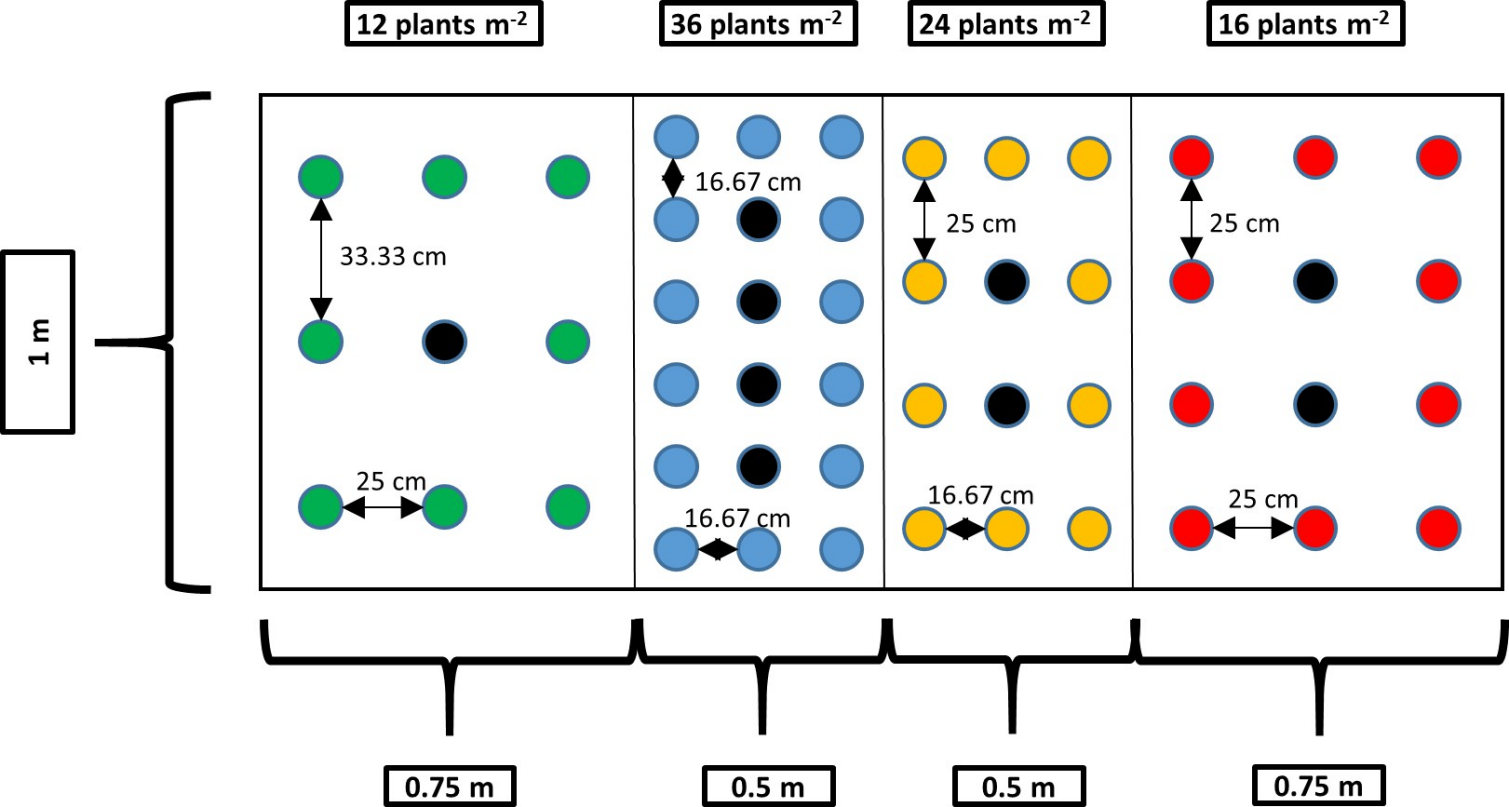
Setup on a greenhouse table for the four densities tested in the density trial, exemplary for replicate 1. The black rectangle represents the table. The colored circles show the positions of the plants. Black circles show the center plants that were used for measurements.

In this trial, four PDs were compared (12, 16, 24, and 36 plants m^-2^). The trial was set up as a randomized complete block design, on three greenhouse tables measuring 2.5 × 1 m^2^, with the four densities randomly arranged on each table (S1 Fig). The greenhouse tables were aligned one behind the other from the west to the east side of the greenhouse compartment, resulting in a continuous experimental area of 7.5 m^2^. They were divided into four plots for the arrangement of the densities per table. The plot length corresponded to the complete table width (1 m). The plot size was 0.75 m^2^ for the PD of 12 and 16 plants m^-2^ and 0.5 m^2^ for the densities of 24 and 36 plants m^-2^ (Fig 1). In each plot, the plants were arranged in three rows (parallel to the table width), with three plants per row at the density of 12 plants m^-2^, four plants per row for the densities of 16 and 24 plants m^-2^, and six plants per row at the density of 36 plants m^-2^. The planting distance was chosen to achieve the desired PD per m^2^ and to maintain half the planting distance between the border plants and the plot border on each side. All non-destructive measurements were taken exclusively on the center plants per plot, meaning that all plants from the two border rows, as well as the first and last plants of the middle row, were excluded. Therefore, the number of center plants differed between the PD treatments. Plants were kept for three weeks under long-day conditions before initiation of flowering. The only destructive measurement was performed on all center plants per plot at the end of the trial at the final harvest 56 days after flower induction. To minimize movement of center plants, the first plants in each row were used to determine the daily water requirement. Average irradiation during the vegetative phase was 174 ± 87.7 μmol m^-2^ s^-1^ at an average temperature of 23.8 ± 3.78°C and a relative humidity of 55.2 ± 21% (mean ± standard deviation of logged values). During the generative phase, irradiation increased with plant height from 157 μmol m^-2^ s^-1^ at initial plant height at the beginning of flowering to 450 μmol m^-2^ s^-1^ when the plants had reached their final height. The average temperature in the dark room was 26.0 ± 4.1°C and the relative humidity was 64.7 ± 22.3%.

### 2.3 Effect of altered length of vegetative growth (V-trial)

**Fig 2:**
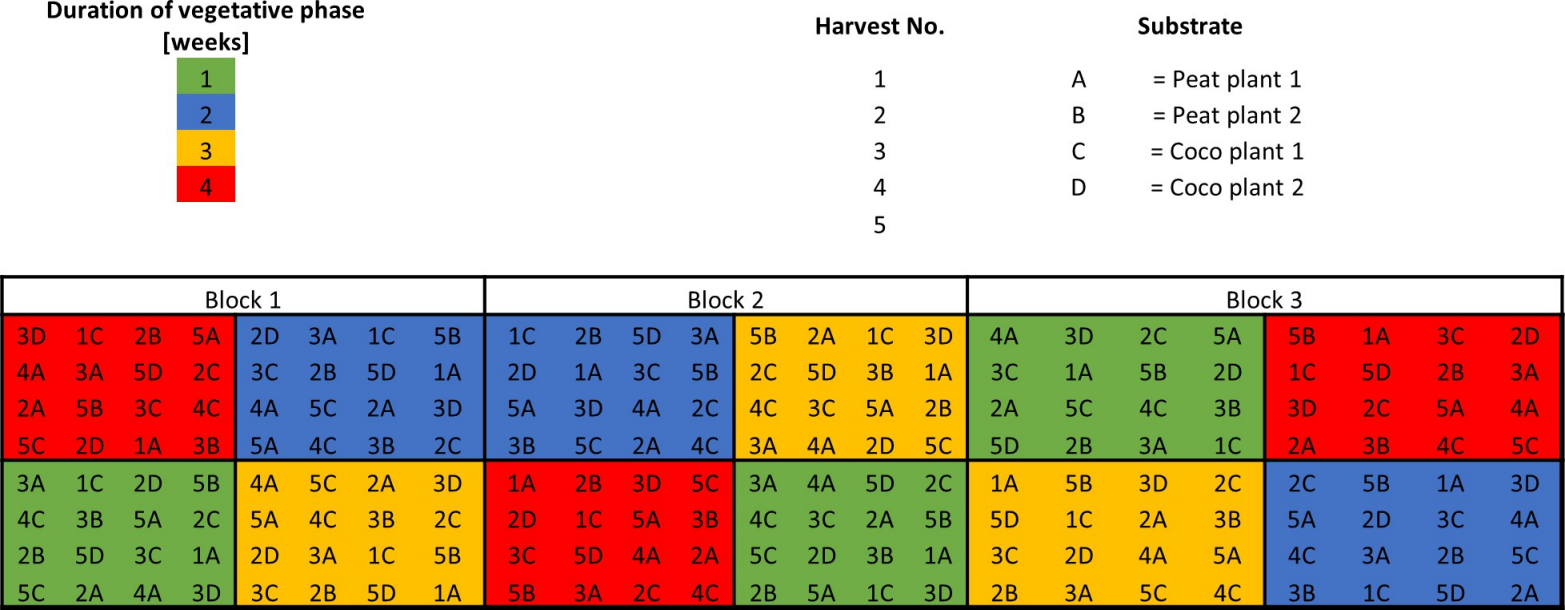
Experimental design for the trial to test the effects of the duration of the vegetative phase (V-trial).

This trial compared four levels of DVP (1, 2, 3, and 4 weeks). The trial was a split-plot design with three replicates, each corresponding to a 2 × 2.5 m^2^ greenhouse table. The greenhouse tables were aligned one behind the other from the west to the east side of the greenhouse compartment, resulting in a continuous trial area of 15 m^2^. Each replicate consisted of four contiguous main plots, each containing 16 plants (four rows and four columns) of the same DVP in a PD of 17 plants m^-2^. The sub-plot factors in this trial were the two substrates used and the five harvest dates (0, 20, 35, 49, and 61 days after flower induction). Harvest date 0 days after flower induction refers to the end of the vegetative phase for all treatments. The substrate was introduced as an additional factor to consider the possible effects of the specific properties of the substrate in combination with the constant pot size and the concentration of the slow-release fertilizer for all tested DVPs. Therefore, peat and coco mixtures were selected, which are commonly used as substrate mixtures in the cannabis industry. At harvest dates 0 and 49 days after flower induction, a single plant per substrate-main plot combination was harvested. On the other three dates, two plants per substrate and main plot were harvested resulting in a total of eight plants per substrate and 16 plants per main plot. Treatment combinations were allocated to plants in each main plot according to a Graeco-Latin square design using harvest date as one factor with four levels (20, 35, 61, and 0/49 days after flower induction) and the combination of plant number and substrate as the second factor (Fig 2).

One of the two plants per plot and substrate earmarked for the final harvest was designated as the scoring plant for the weekly non-destructive measurements (B-plant) and daily weighing for determination of water content, but was also included in the final destructive measurement at the final harvest. During the vegetative phase, the average irradiation was 208 ± 52.7 μmol m^-2^ s^-1^, at an average temperature of 24.1 ± 3.6°C and a relative humidity of 42.7 ± 22.7%. When the experiment was moved to the dark room with only artificial lighting, care was taken to ensure that each plot was illuminated by exactly one CHD Agro 400 lamp, which was mounted centrally above the plot surface. The distance from the lamp to the top of the canopy was set separately to 90 cm for each plot at the beginning of flowering and was readjusted after three weeks into flowering when height growth terminated. This ensured that the average irradiance at the top of the canopy was ~400 μmol m^-2^ s^-1^ for all treatments during the flowering phase. For further details regarding the trial setup, we refer to [31].

### 2.4 Data collection

#### 2.4.1 Light and non-destructive measurements

The non-destructive measurements comprised the measurement of height from the stem base to the plant tip (tip of the apical meristem on the main stem) as well as the number of nodes on the main stem and the cumulative length of lateral shoots. For this purpose, measurements were taken from the shoot base on the main stem to the apical meristem of each lateral shoot. In addition, the relative interception of the canopy of incoming light within the photosynthetic active range (400–700 nm) was measured in both trials using an Accupar LP-80 ceptometer (Decagon devices, Hopkins Pullmann Court, USA) as the ratio of light intensity within canopy at pot height (I_p_) to intensity of incoming light (I_o_). The term light interception will be further used to describe the measurement of the intercepted photosynthetic active radiation. I_p_ was measured at pot height between rows. Plot-specific average values (D-trial: *n* = 2; V-trial: *n* = 3) were calculated and used for statistical analysis. Measurements were taken as part of the weekly non-destructive measurements in the D-trial and only once in the V-trial at the end of the vegetative phase, before the first harvest. In the V-trial, I_o_ represents the plot-specific average value (*n* = 3) of light intensity at the top of the canopy, measured above each row. In the D-trial, a single value of I_o_ for the whole cultivation area was calculated as the mean value of a 20 × 20 × 20 cm^3^ grid measurement at 16 cm above table height in the empty darkroom (without plants) using a FLAME-S-XR1-ES point spectrometer (Ocean Optics Germany GmbH, Ostfildern, Germany). In addition, the R:FR ratio at mid-canopy height and pot height was determined in the D trial. For this purpose, the value of the R:FR ratio was recorded at ten evenly distributed points per plot between the rows of plants at mid-canopy height and pot height using the FLAME-S-XR1-ES point spectrometer. The mean value was then calculated so that one value for R:FR ratio at mid-canopy height and one at pot height were used per plot for the subsequent statistical analysis. The measurements of the R:FR ratio were conducted once at 35 days after flower induction.

#### 2.4.2 Gas exchange measurements

The maximum photosynthetic rate (A_max_) was measured with a portable gas exchange fluorescence system GFS-3000 (Walz, Effeltrich, Germany) in the D-trial. The measurements were carried out on the foremost center plant in each density plot. For each plant, the uppermost fully developed leaf on the main stem and the uppermost leaf on the main stem of the lower half of the plant were measured. To do this, the plant height was first determined and the first leaf directly below the half mark was selected. The standard 3010-S measuring head with a 4 cm^2^-cuvette was used for the measurements and light was provided using a 3056-FL LED array/PAM fluorometer. The settings in the cuvette during the measurements were 2000 μmol m^-2^ s^-1^ for the light intensity, 400 ± 1 ppm for the CO_2_ concentrations, 30 ± 0.5°C for the leaf temperature, and 50 ± 1% for the relative humidity. The air flow rate was set to 750 μmol min^-1^. Each leaf was acclimatized in the cuvette for 15 minutes before the reading was recorded.

#### 2.4.3 Destructive measurements

For all destructive measurements, the plants were cut off at the base of the stem just above the substrate. The plant organs were then separated into stems, leaves (without petiole), and inflorescences. The leaf area was determined immediately using a LI-3100 Area Meter (LI-COR, Lincoln, NE, USA). Note that in the V-trial the leaf area was only measured up to the onset of leaf senescence (this corresponds to the first three harvests). At the final harvest, the plants were divided into their upper and lower halves according to their height and all organs were separated according to which half of the plant they belonged to. B-plants in the V-trial were only separated into vegetative and generative biomass. Leaf and stem material was dried in a drying oven at 60°C for 48 h before the dry mass was determined. Inflorescences were separated into a total of three fractions: apical inflorescences of the main stem (MAI) and axillary inflorescences of lateral side shoots, each for the upper and lower half of the plant. Note that by definition MAI does not occur in the lower half of the plant. The number of inflorescences harvested was counted for each fraction. Inflorescence material was air-dried in a dark tent at ~20°C and a humidity of 50–60% for 14 days before the dry mass was determined. The average mass per inflorescence for each fraction could then be calculated from the total yield per fraction divided by the number of inflorescences per fraction. By definition, the number of MAI was always one. Thus, the total yield of this fraction equals the average inflorescence mass.

#### 2.4.4 Chemical analysis

For the chemical analysis, the inflorescences were ground using a knife mill GRINDOMIX GM 200 (Retsch, Haan, Germany). The cannabinoid extraction was performed according to the method of [32], with slight modifications. Two technical replicates were analyzed per sample. For this purpose, 100 ± 10 mg of the ground samples were weighed into 100 mL volumetric flasks for each technical replicate. For the extraction, 100 mL of a methanol 90%/Chloroform 10% mixture (v/v) was added and the volumetric flasks were placed in an ultrasonic bath (40°C) for 30 min. After subsequent cooling to room temperature, the solution was filtered through 0.45 μm polytetrafluoroethylene syringe filters. The first 2 mL of the sample that passed through the filter was discarded before the solution was transferred to HPLC vials, which were stored at -20°C until injection into the HPLC. On the day of extraction, the residual moisture was determined for each sample using a moisture analyzer DBS 60–3 (Kern and Sohn GmbH, Balingen, Germany). The HPLC system used for cannabinoid analysis (1290 Infinity II LC System, Agilent, Santa Clara, CA, USA) was equipped with a quaternary pump and an autosampler, which was cooled down to 4°C for the duration of the sample runs. The detector was a diode array spectrophotometer and the detection wavelength was 230 nm. The HPLC analysis followed the method described by [30]. The chromatographic separation was performed on a Nucleosil 120–3 C8 column (125 mm × 4 mm i.d., 3.0 μm) with a guard column EC 4/3 Nucleosil 120–3 C8 (Macherey-Nagel, Oensingen, Switzerland) with HPLC-grade methanol (solvent A) and 0.1% acetic acid in HPLC-grade distilled H_2_O (solvent B; Sigma-Aldrich, Saint Louis, MO, USA) at a constant flow rate of 0.7 mL min^−1^ with gradient elution mode. CBD and CBDA concentrations were calculated using analytical reference standards for CBD (C-045) and CBDA (C-144) (Sigma-Aldrich, Darmstadt, Germany). The calculated concentrations were corrected for the measured residual moisture content. The CBD concentrations presented correspond to the weighted sum of measured CBD and CBDA concentrations. The CBDA concentration was therefore multiplied by the value 0.877 to obtain the corresponding value as CBD equivalent, which was added to the measured CBD concentration.

### 2.5 Statistical analysis

#### 2.5.1 Logistic function

To describe the measured light interception over the cultivation time in the D-trial as a function of the PD, the logistic growth function was used for repeated data of each plot across time points *t*:

$${FI}_{tij}=\frac{{L_{max}}_{ij}}{1+e^{-k_{ij}*(t-t_{{half}_{ij}})}}+e_{tij}$$ (1)

where *FI*_*tij*_ is the fraction of light intercepted at time point *t* of block *i* in density *j*. The term ${L_{max}}_{ij}$ describes the asymptotic maximum fraction of the intercepted light of the density *j* in block *i* and *t* is the time point of measurement in weeks. The parameter *k*_*ij*_ is the plot-specific steepness of the curve and $t_{{half}_{ij}}$ is the plot-specific time point, when 50% of ${L_{max}}_{ij}$ is reached and *e*_*tij*_ is the error of *FI*_*tij*_ with plot-specific variance.

The parameter estimates and their standard error were then forwarded to the second stage and analyzed for significant differences between PD using the following model:

$${ȳ}_{ij}=\mu +b_{i}+{б}_{j}+f_{ij}$$ (2)

where ${ȳ}_{ij}$ is the estimated parameter of the *i*-th block and the *j*-th density. The parameters *b*_*i*_ and б_*j*_ are the fixed effects for the *i*-th block and the *j*-th density, respectively. The term *fi*_*j*_ is the residual error for the parameter estimate ${ȳ}_{ij}$ and has a variance that is proportional to the variance of the parameter estimate. In the case of a significant density factor, mean parameter estimates for each density were calculated. Otherwise, a single mean parameter was estimated and used to determine the final curves.

#### 2.5.2 Regression analysis

Linear regression models for all measured plant traits from the destructive and non-destructive measurements on PD and DVP were fitted. A lack-of-fit test was performed beforehand for all models to confirm the linear relationship between the regressor and response variable. If the lack-of-fit test resulted in significant deviations from linearity, PD or DVP were treated as qualitative, and analysis was based on mean comparisons. Otherwise, the variable was treated as quantitative. For most traits on a single-plant basis, the regression was additionally fitted depending on different positions within the plant. This involves, for both trials, the parameters of leaf and stem dry mass, dry yield, and CBD yield, separately for the upper and lower plant half. Likewise, CBD concentration and average inflorescence mass for different inflorescence positions were analyzed this way. In the D-trial, A_max_, SLA, and R:FR ratio were additionally analyzed, taking into account position effects.

For this purpose, the following model was used for the linear regressions in the D-trial:

$$y_{iklm}=\mu +b_{i}+\rho_{k}+{(\beta }_{0}+\beta_{k})*d_{iklm}+{p_{ikl}+e}_{iklm}$$ (3)

where *y*_*iklm*_ is the observation of the *i*-th block and the *l*-th plot at the *k*-th position of the *m*-th plant, *b*_*i*_ is the fixed effect of the *i*-th block, and μ and *β*_0_ correspond to the common intercept and common slope term on density *d*_*iklm*_ at the *k*-th position of the *m*-th plant in *l*-th plot of the *i*-th block, respectively. The term *ρ*_*k*_ is the fixed main effect of the *k*-th position and *β*_*k*_ the position-specific slope. The term *p*_*ikl*_ for the random effect of position *k* in the *l*-th plot of the *i*-th block. The random error of *y*_*iklm*_ is denoted by *e*_*iklm*_. For position effects of the same plot and plant, a first-order autoregressive variance-covariance structure with heterogeneous variance was assumed. For qualitative factor PD, ${(\beta }_{0}+\beta_{k})*d_{iklm}$ in (3) were replaced by *τ*_*n*_+(*τρ*)_*kn*_ for the *n*-th PD.

The following model was used in the V-trial for traits measured at the final harvest in dependence on position:

$$y_{ijklm}=\mu +b_{i}+{б}_{j}+\rho_{k}+{\left(б\rho \right)}_{jk}+(\beta_{0}+\beta_{j}+\beta_{k}+\beta_{jk})*l_{ijklm}+p_{ijkl}+e_{ijklm}$$ (4)

where *y*_*ijklm*_ is the observation of the *i*-th block of the *j*-th substrate at the *k*-th position of the *m*-th plant in the *l*-th plot. The fixed main effects for the *j*-th substrate and the *k*-th position as well as their corresponding interaction effects are denoted by б_*j*_, *ρ*_*k*_, and (б*ρ*)_*jk*_, respectively. The terms *b*_*i*_, μ, *p*_*ijkl*_ and *e*_*ijklm*_ are analogously defined as in model (3). *β*_0_, *β*_*j*_, *β*_*k*_, and *β*_*jk*_ are the common, substrate, position, and substrate-by-position specific slopes DVP at the *i*-th block of the *j*-th substrate at the *k*-th position of the *l*-th plot (*l*_*ijklm*_). Again, a first-order autoregressive variance-covariance structure with heterogeneous variances was fitted for the position effects of the same plant. For position effects of the same plot, the structure is extended by fitting an addition constant covariance. For qualitative factor duration $(\beta_{0}+\beta_{j}+\beta_{k}+\beta_{jk})*l_{ijklm}$ in (3) were replaced by $\vartheta_{o}+{\left(\vartheta б\right)}_{jo}+{\left(\vartheta \rho \right)}_{ko}+{\left(\vartheta б\rho \right)}_{jko}$ for the o-th DVP.

For traits from the weekly non-destructive measurements, which were only measured once per plant, models (3) & (4) were altered by replacing position with timepoint. Leaf area per plant, No. of inflorescences per plant, the relative fractions of leaves, stems, and inflorescences on total plant biomass, and area-based parameters (total biomass m^-2^, LAI, yield m^-2^, CBD yield m^-2^) were also estimated without position effect. Hence, model (3) was simplified to analyze these traits for the D-trial, and all effects involving position were dropped from the model.

In the V-trial, these traits were measured over several harvest time points. This was taken into account in the statistical analysis. Hence, model (4) was adjusted by replacing the effects for the position with the timepoint of harvest. As the timepoint of harvest was accounted for in the row-column design of each plot, random row and column effects within plots were added to the model. For CBD yield m^-2^ and No. of inflorescences, all effects involving harvest were dropped from the model, as these traits were only measured at the final harvest.

All results are presented for the latest time point of measurement, which coincides with the timepoint of the final harvest for all plant traits, except leaf area per plant and LAI in the V-trial. These were only measured until the third harvest, hence, results are presented for the third harvest. In the V-trial, interaction effects were either not significant or interactions were not relevant in the sense that the relative order of position and /or harvest changed depending on substrate. We, therefore, presented means across substrates in all cases to simplify the presentation. For the D- and V-trial, the resulting F-test tables and results of lack-of-fit tests for each analyzed plant trait are provided in the supplementary material (S2 and S3 Tables).

For all statistical models, a position-specific heterogeneous error variance was allowed if this resulted in a better model fit. For the final analysis of each plant trait, all non-significant effects were removed from the model. Normal distribution and variance homogeneity were checked graphically using the residual plots. The analysis was conducted using proc nlin for fitting of logistic function and proc mixed for the mixed models using the statistical software SAS version 9.4 (The SAS Institute, Cary, NC, USA). The raw data from both the D-trial (S1 Data) and the V-trial (S2 Data) are provided.

## 3. Results

### 3.1 Plant morphology

**Fig 3:**
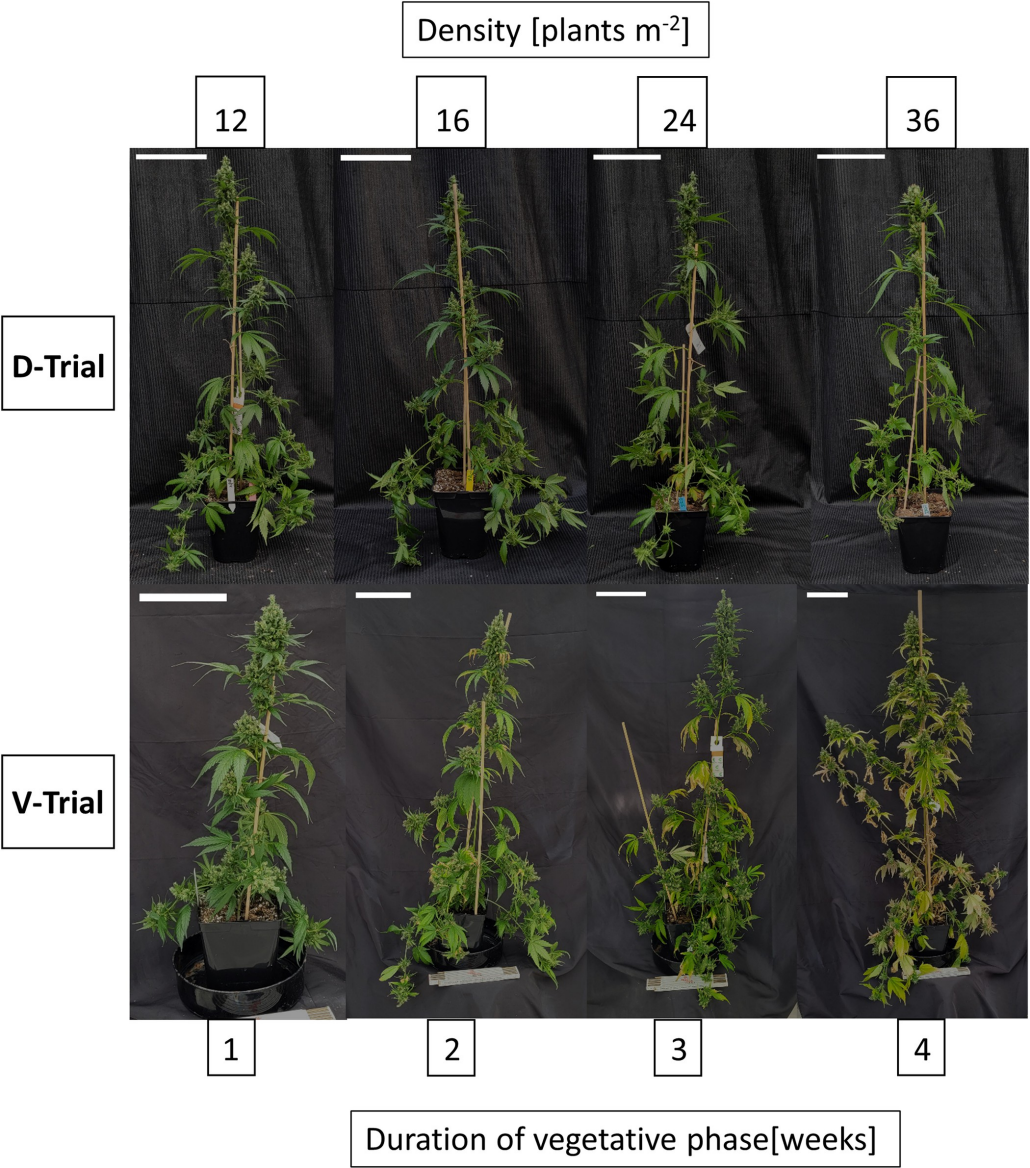
Exemplary plants at final harvest for the density trial (D-trial) trial of increasing duration of vegetative growth phase (V-trial). Scale = 15 cm.

DVP led to clearly recognizable visual effects on the plants. As expected, the extension of DVP was accompanied by taller plants, longer side shoots, and a larger number of main shoot nodes and inflorescences. Leaf senescence was more advanced in plants with a longer DVP. The morphological effects were less clear when comparing PD. Plants from higher densities tended to be slightly taller and narrower (Fig 3).

The visual impressions are supported by linear regressions on the two test variables from the D- and V-trial. The lack-of-fit test was not significant for any of the target variables leaf area, plant height, cumulative lateral shoot length, number of nodes, and number of inflorescences in both trials. Therefore, the linear regression model is proposed as a suitable model (S2 Table). In the V-trial, all slope terms for the respective target variables significantly differed from zero and showed a positive correlation with the DVP (Table 1). Consequently, the plants with a vegetative phase of four weeks had the highest maximum leaf area (4534 ± 155 cm^2^ plant^-1^) at the third harvest. This was reduced by 77.4% in the shortest DVP. Likewise, plant height (100.7 ± 1.4 cm), accumulated lateral shoot length (496.8 ± 36.5 cm), number of nodes on the main shoot (24.8 ± 1.0), and number of inflorescences (143.0 ± 18.6) were the highest for plants with a DVP of four weeks and were reduced by 35%, 61.8%, 28.5%, and 55.8%, respectively, in the shortest DVP (one week).

**Table 1:** Estimated slopes of the linear regression of target traits as a function of plant density (D-Trial) and length of the vegetative phase (V-Trial).

Trial	Leaf area [cm^2^ plant^-1^]	Height [cm]	Shoot length [cm]	No. Nodes	No. Inflorescences
D [plants m^-2^]	- 9.30	0.29*	-0.42	-0.03	-0.16
V [weeks]	1134.56*	11.84*	108.11*	2.08*	28.60*

Leaf area refers to the measurement at the final harvest for D-trial and maximum value for V-trial as measured at the third harvest (35 days after flower induction). Values marked with * indicate slope terms significantly different from zero at α = 0.05.

Note that for height and lateral shoot length, the interaction of substrate and slope term was also significant, wherefore a separate slope term for each substrate was estimated. The values (Table 1) represent the mean value for both substrates. According to the regression analysis, only plant height was positively associated with PD in the D-trial. The tallest plants, with 82.25 ± 1.57 cm, were found in the highest plant density of 36 plants m^-2^, while they were 10.4% smaller in the lowest density. The slope terms for lateral shoot length (*p* = 0.53), number of nodes (*p* = 0.20), and number of inflorescences (*p* = 0.63) were not significantly different from zero. Therefore, the mean values for these target variables were independent of plant density with estimated intercepts at 282.68 ± 16.94 cm (shoot length), 17.62 ± 0.65 (number of nodes), and 77.81 ± 8.89 (number of inflorescences). Similarly, for leaf area, no significant effect of PD was found (p = 0.07). Hence, a common intercept of 1491.8 ± 48.9 cm^2^ plant^-1^ for all tested PD was estimated.

### 3.2 Biomass allocation between plant organs and plant section

**Fig 4:**
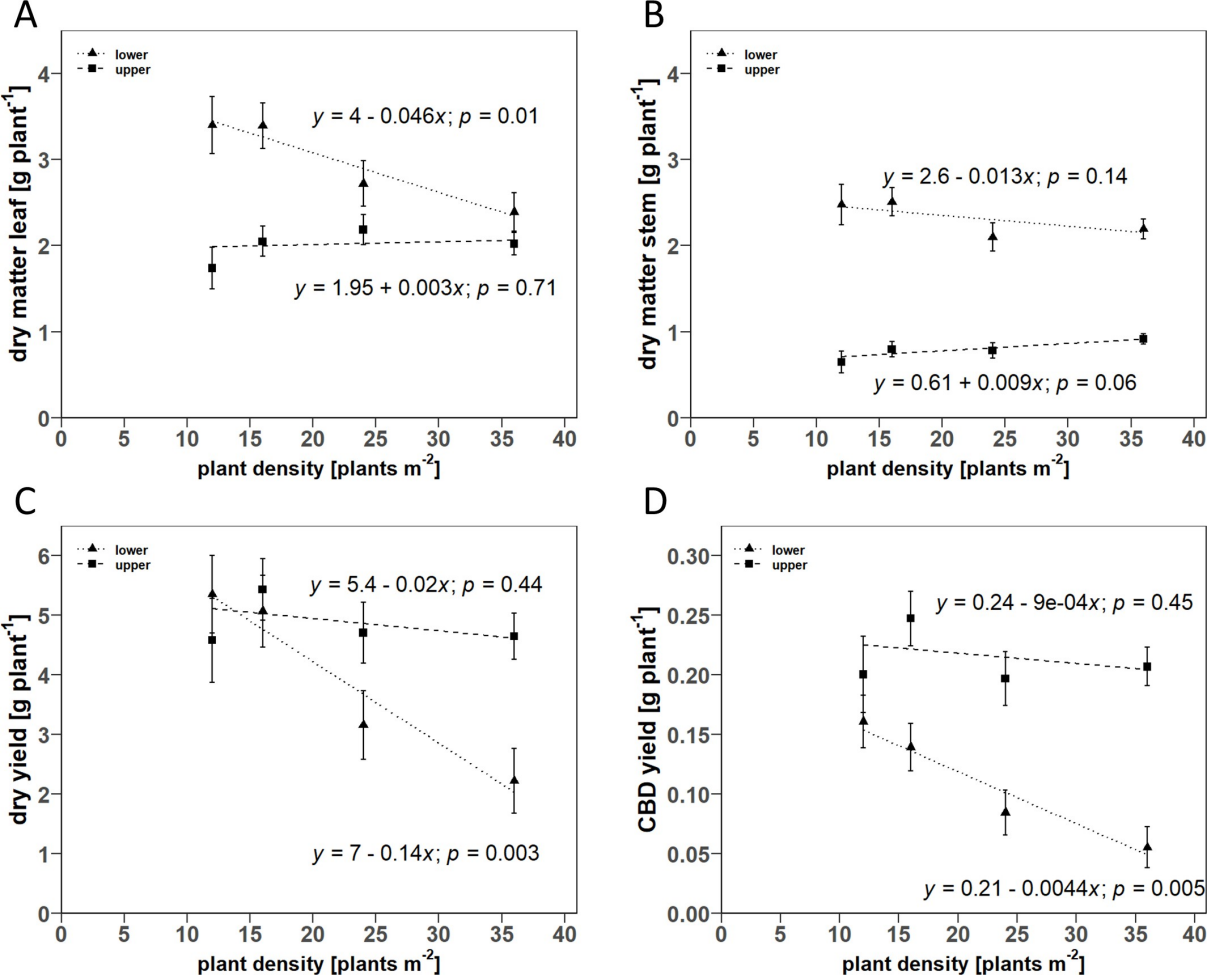
Scatter plots of the linear regression for the relationship between plant density and A) dry leaf matter, B) dry stem matter, C) dry yield, and D) CBD yield in dependence on the position at the upper and lower plant half. Point symbols indicate the mean values for the respective plant halves. Error bars indicate the estimated standard error of the mean (*n* = 3). Dashed lines show the fitted linear regression models for each plant half. *p*-values indicate wether the estimated slope term was significantly different from zero.

The lack-of-fit test for the linear regression of leaf and stem dry matter, dry yield (dry matter of inflorescences), and CBD yield in the dependence of upper and lower plant half indicated that the linear model satisfactorily described the results for the D- and V-trial. In the D-trial, the F-test for the specific slope term for the plant half was significant in all cases. Therefore, separate regression lines were estimated for the upper and lower half of the plant. A positive relationship with PD was estimated for leaf and stem mass of the upper half of the plant. However, in both cases, the estimated slopes were not significantly different from zero. In contrast, this relationship was negative for the lower half of the plant with a slope of -0.046 g (*p* = 0.01) and -0.013 g (*p* = 0.14) per additional plant m^-2^ for leaf and stem mass, respectively (Fig 4). Consequently, the ratio of upper to lower dry mass of leaves and stems shifted from 0.51 and 0.26, respectively, in the lowest PD to 0.85 and 0.42, respectively, in the highest PD. There was also a significant negative correlation between dry and CBD yield of the lower half of the plant with PD with estimated slopes of -0.14 g (*p* = 0.003) and—0.0044 g (*p* = 0.005) per additional plant m^-2^, respectively. For the upper half of the plant, correlations with PD were also negative. Still, the estimated slopes for dry (-0.02 g) and CBD yield (-0.0009 g) per additional plant m^-2^ were not significantly different from zero. Consequently, the proportion of dry yield of the lower half of the plant decreased from 53.8% to 32.3% from the lowest to the highest PD, and the proportion of CBD yield also decreased from 44.5% to 21.1%.

**Fig 5:**
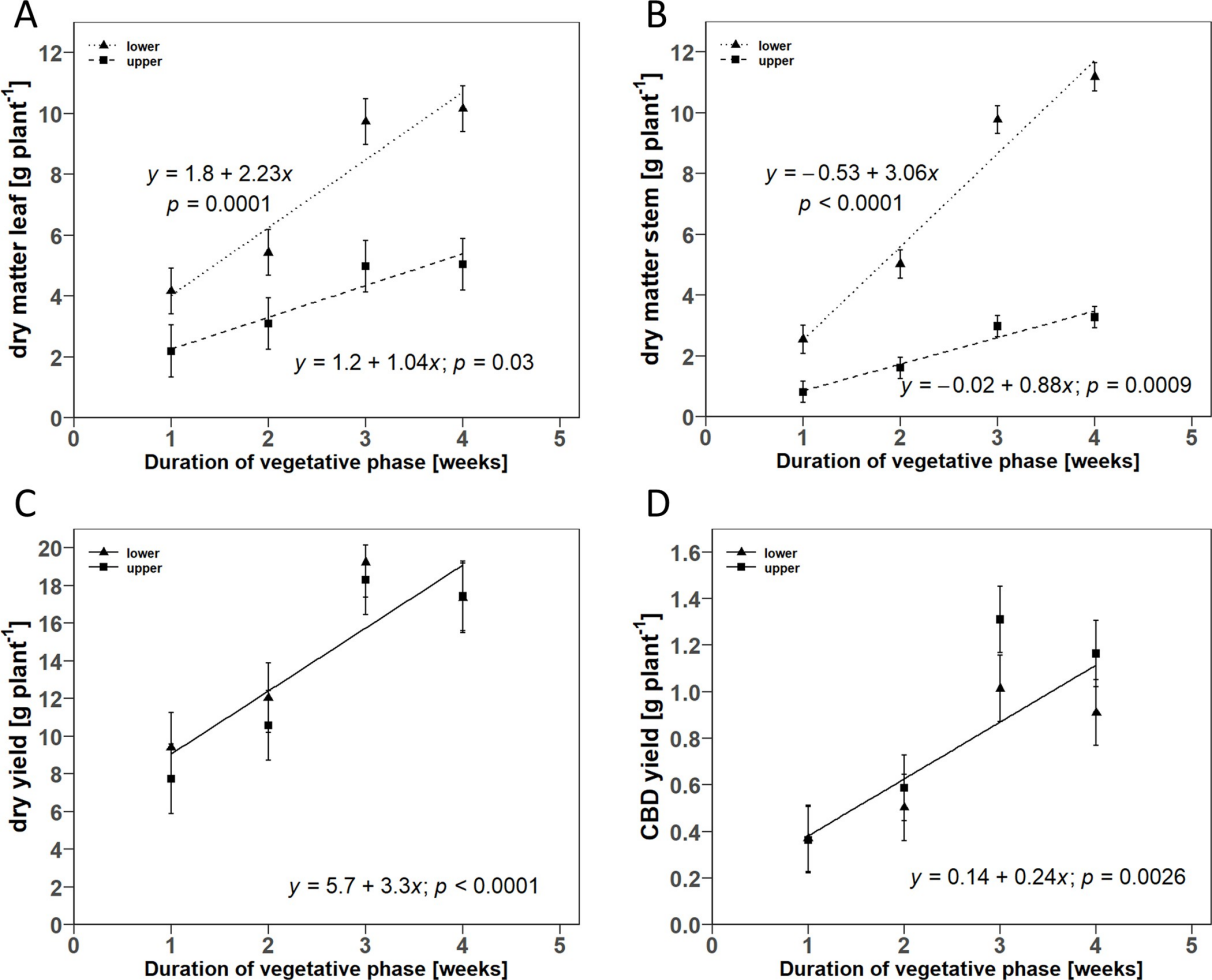
Scatter plots of the linear regression for the relationship between duration of vegetative phase and A) dry leaf matter, B) dry stem matter, C) dry yield, and D) CBD yield in dependence on the position at upper and lower plant half. Point symbols indicate the mean values for the respective plant halves. Error bars indicate the estimated standard error of the mean (*n* = 3). Dashed lines show the fitted linear regression models for each plant half. *p*-values indicate wether the estimated slope term was significantly different from zero.

In the V-trial, both leaf and stem dry matter were positively associated with increasing DVP for both plant halves. All estimated slopes were significantly greater than zero, with the estimated slopes for the lower half of the plant being greater for leaf and stem dry mass than for the upper half of the plant (Fig 5). Consequently, the proportion of leaf mass of the lower half of the plant to the total leaf mass varied only minimally between 63.7% and 66.8% within the tested DVP. Similarly, the lower stem dry mass proportion varied between 75.8% and 77.4%. The mean dry yields in the V-trial varied between 17.1 and 37.5 g and showed a significant positive linear correlation with DVP (slope = 3.3 g week^-1^). The F-test of the fitted linear regression model was not significant for the position, so a common linear regression equation was estimated for the upper and lower plant half. Similarly, a common linear regression equation was also estimated for CBD yield, showing a positive linear relationship with DVP (slope = 0.24 g week^-1^; Fig 5).

**Fig 6:**
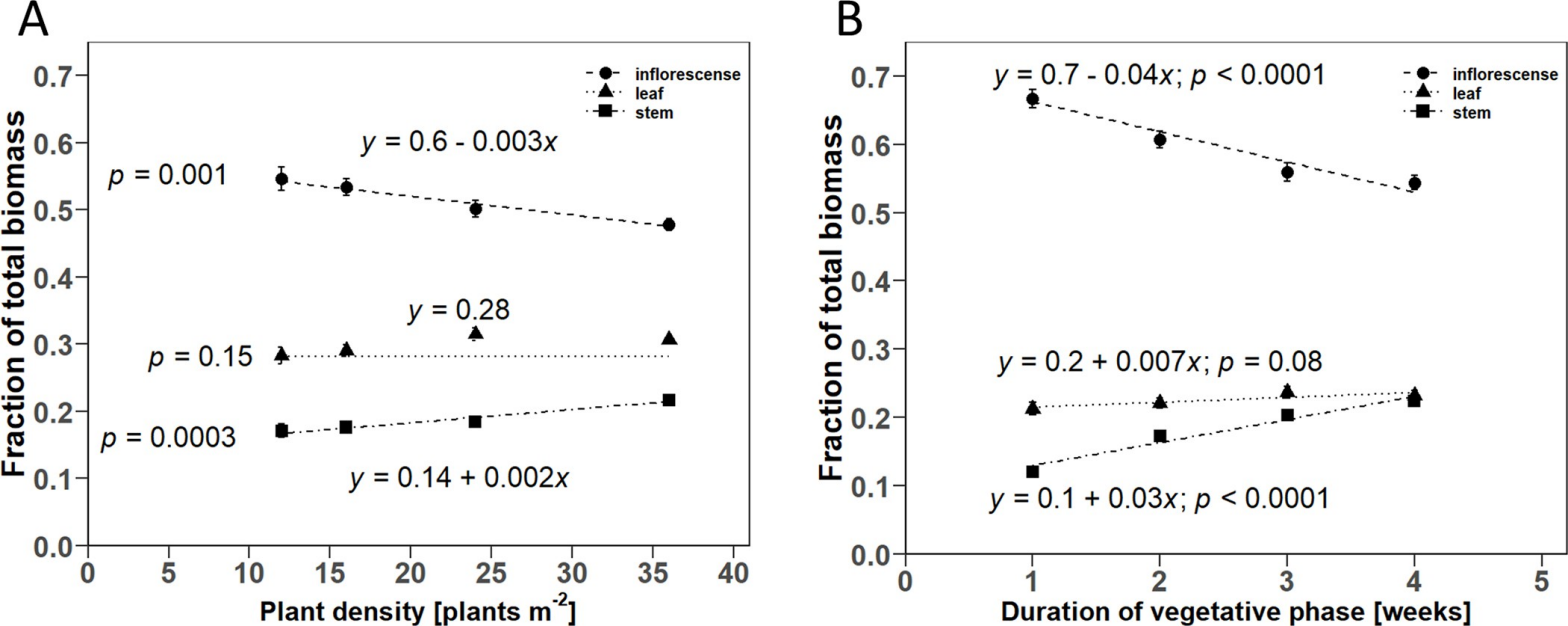
Scatter plots of the linear regression for the relationship between the proportion of individual plant organs in the total biomass with A) plant density and B) duration of the vegetative phase. Point symbols indicate the mean values for the respective plant organs. Error bars indicate the estimated standard error of the mean (*n* = 3). Dashed lines show the fitted linear regression models for each plant organ based on the separate regression analyses. For A): *p*-values result from the global F-test for the fixed effect of plant density. For B): The *p*-values indicate whether the estimated slope terms of the final harvest are significantly different from zero.

In both the D-trial and the V-trial, there was a significant linear influence on the fraction of the individual plant organs on the total plant dry mass. The inflorescences generally made up the largest proportion of all plant organs. In the D-trial, the proportion of inflorescence mass to total biomass decreased from 54.6% to 47.8% from lowest to highest PD. The estimated slope of the linear regression was -0.3% per additional plant m^-2^. In contrast, the stem proportion increased by 0.2% per additional plant m^-2^ (Fig 6A). The regression analysis for the leaf proportion of the total biomass showed no significant influence of the PD, wherefore the regression equation only consists of the estimated intercept of 28.2 ± 1%. In the V-trial, the proportion of inflorescence mass in the total mass decreased from 66.7% to 54.4% from shortest to longest DVP. The estimated slope of the linear regression was -4.4% per week (Fig 6B). A positive slope of 3.4% per week was found for the proportion of stem mass. The proportion of stem mass increased from 12% to 22.4% from shortest to longest DVP. The leaf proportion only increased marginally from 21.3% to 23.2% from shortest to longest DVP. Although the global F-test showed a significant influence of DVP depending on the harvest, the estimated slope at the final harvest was not significantly different from zero.

### 3.3 Intra-plant gradient of CBD concentration and average inflorescence mass

The linear regression for the relationship between CBD concentration and average inflorescence mass as a function of inflorescence fraction showed no influence of PD on these two parameters. However, the effect of fraction was significant in both cases. The estimated intercepts (Table 2) showed a gradient of CBD concentration from MAI (4.91%) > axillary inflorescence upper (4.16%) > axillary inflorescence lower (2.65%). The same order is shown for the average inflorescence mass, which was highest for MAI (1.47 g inflorescence^-1^) and lowest for the axillary inflorescences of the lower half of the plant (0.07 g inflorescence^-1^).

**Table 2:** Estimated intercepts of the regression analysis for CBD concentration and average inflorescence mass in dependence on inflorescence fractions (intercept) in the D-trial.

Fraction	CBD concentration [%]	Avg. mass [g inflorescence^-1^]
MAI	4.91 ± 0.09	1.47 ± 0.08
upper	4.16 ± 0.08	0.22 ± 0.02
lower	2.65 ± 0.10	0.07 ± 0.01
*p*-value		
Density [D]	0.44	0.17
Fraction [F]	<0.0001	<0.0001
Interaction D × F	0.21	0.71

Inflorescence fractions were the main apical inflorescence of the main shoot (MAI) and axillary inflorescences of the upper and lower plant half. Results are presented as estimated intercept ± estimated standard error. The *p*-values result from the global F-test for the fixed effects of plant density, inflorescence fraction, and their interaction.

**Fig 7:**
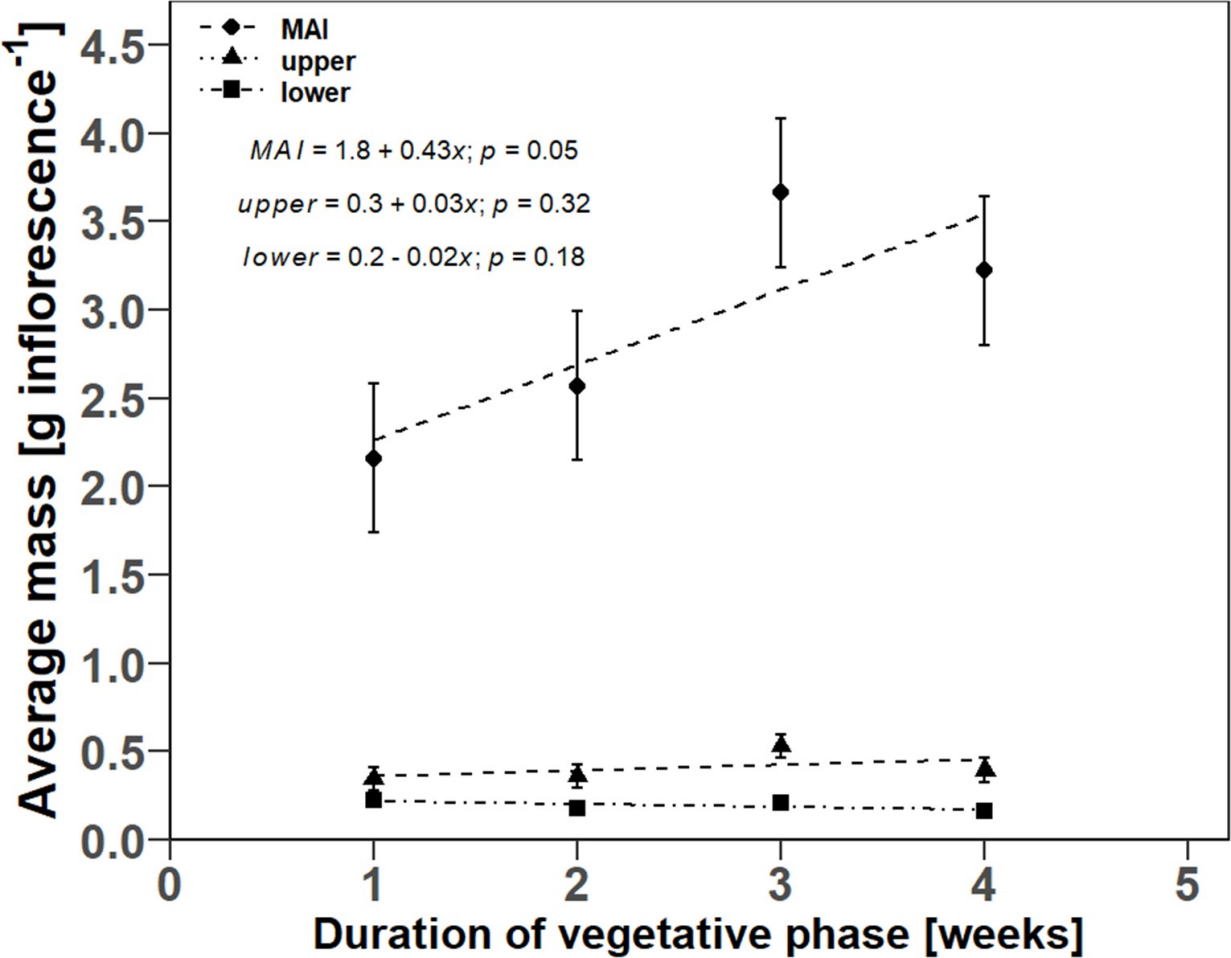
Scatter plots of the linear regression for the relationship between the duration of the vegetative phase and the average mass of single inflorescences. Point symbols indicate the mean values for the respective plant organs. Error bars indicate the estimated standard error of the mean (*n* = 3). Dashed lines show the fitted linear regression models for the inflorescence fractions of the main apical inflorescence (MAI) and axillary inflorescences of the upper and lower plant half. The *p*-values indicate whether the estimated slope terms of the final harvest are significantly different from zero.

A linear regression was also found for the relationship between DVP and average inflorescence mass. The average mass of MAI ranged from 2.16 g inflorescences to 3.66 g inflorescences. The estimated slope of the regression line was 0.43 g inflorescence^-1^ week^-1^ (Fig 7). The intercepts for the axillary inflorescences of the upper and lower half of the plant were 0.33 ± 0.08 g inflorescence^-1^ and 0.23 ± 0.03 g inflorescence^-1^, respectively, while in both cases, the estimated slope term was not significantly different from zero.

CBD concentrations achieved in the V-trial were generally higher than in the D-trial. In contrast to the D-trial, the lack-of-fit test of the linear regression model was significant. Hence, DVP was treated as a qualitative variable, and estimated means were compared using pairwise comparisons (Table 3). The CBD concentrations of the MAI were not significantly different from the axillary inflorescences of the upper plant half. The effect of vegetation length was constant for the three inflorescence positions. The CBD concentrations of plants with a 3-week or 4-week vegetative phase did not differ significantly from each other but indicated higher concentrations with 6.6 ± 0.30% (MAI), 7.0 ± 0.36% (upper plant half), 5.25 ± 0.30% (lower plant half) than the plants with a one- or two-week vegetative phase, where CBD concentration was reduced by 18.9%, 28.9%, and 24.4%, respectively.

**Table 3:** Mean CBD concentration (%) for duration of vegetative phase (DVP) and inflorescence fraction.

DVP [weeks]	CBD concentration [%]		
	MAI	upper	lower
1	5.11 ± 0.30^bA^	4.66 ± 0.36^bA^	3.91 ± 0.30^bB^
2	5.54 ± 0.30^bA^	5.3 ± 0.36^bA^	4.03 ± 0.30^bB^
3	6.73 ± 0.30^aA^	7.28 ± 0.36^aA^	5.32 ± 0.30^aB^
4	6.48 ± 0.30^aA^	6.72 ± 0.36^aA^	5.18 ± 0.30^aB^

Inflorescence fractions were the main apical inflorescence of the main shoot (MAI) and axillary inflorescences of the upper and lower plant half. Results are presented as mean values ± estimated standard error (*n* = 3). Means within the same column bearing at least one identical lowercase letter did not differ significantly from each other at α = 0.05 between different DVPs. Means within the same row bearing at least one identical capital letter did not differ significantly from each other at α = 0.05 between inflorescence fractions.

### 3.4 Photosynthesis and light interception

**Fig 8:**
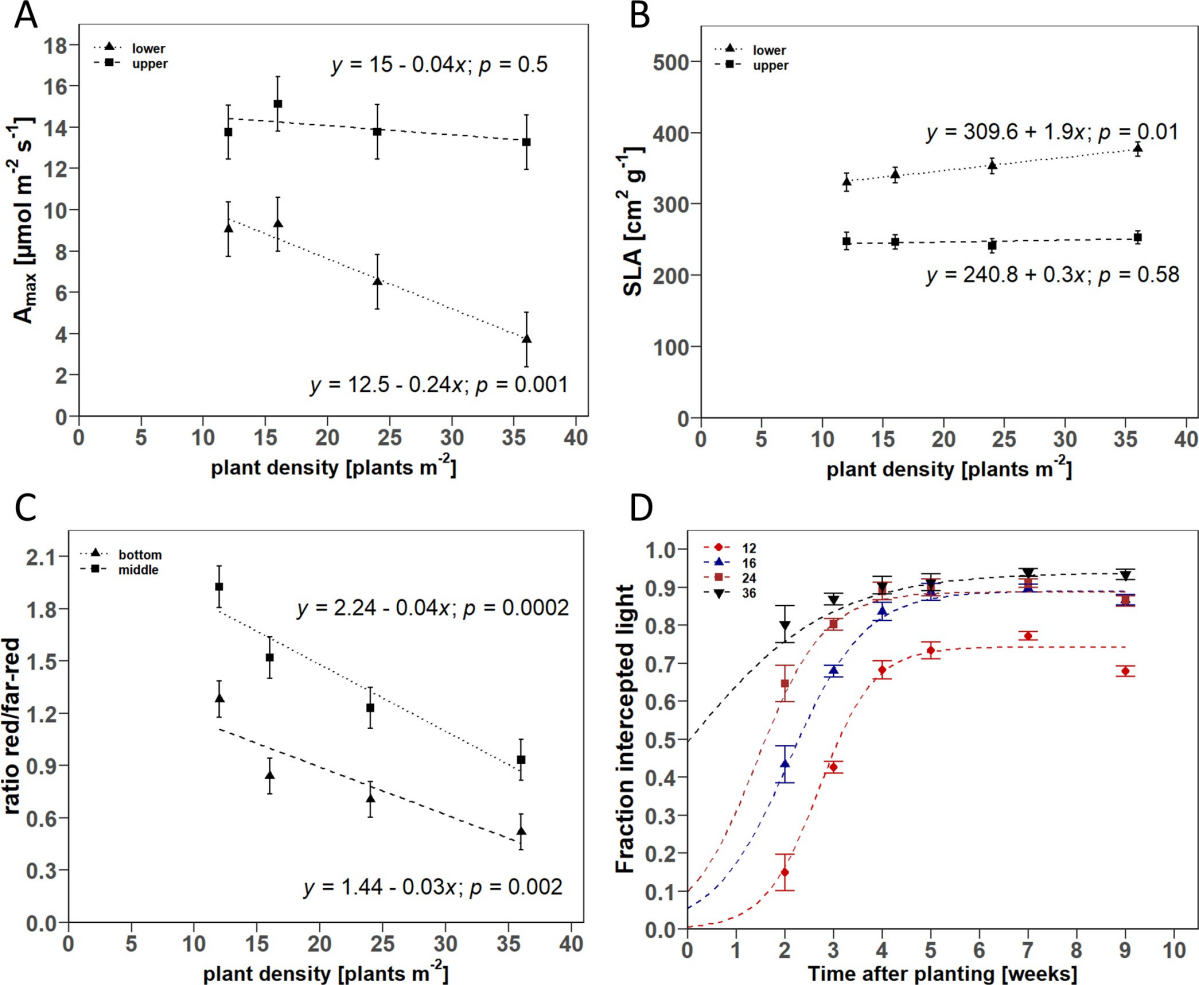
Scatter plots of the relationship between plant density and A) maximum photosynthetic rate (A_max_), B) specific leaf area (SLA), C) red/far-red ratio, and D) fraction intercepted light. Point symbols show the estimated means for the respective plants, grown under densities of 12, 16, 24, and 36 plants m^-2^. Error bars indicate the estimated standard error of the mean (*n* = 3). For A), B), C): Dashed lines show the fitted linear regression models in dependence on A) the uppermost fully developed leaf (upper) and uppermost leaf of the lower plant half (lower), B) leaves of upper and lower plant half, and C) measurements at medium canopy height (middle) and pot height (bottom). The *p*-values indicate whether the estimated slope terms are significantly different from zero. For D): Dashed lines show the fitted logistic functions for the tested plant densities.

For the D-trial, additional linear regressions were estimated for A_max_, specific leaf area (SLA), and R:FR ratio as a function of position in the canopy. A_max_ showed no significant slope in the measurements on the uppermost leaves for increasing PD. In contrast, A_max_ measured on leaves of the lower half of the plant was negatively correlated with PD (Fig 8). The estimated slope of the fitted linear regression equation was -0.24 μmol m^-2^ s^-1^ per additional plant m^-2^. The SLA of the leaves of the lower half of the plant was positively correlated with PD and increased by 1.9 cm^2^ g^-1^ per additional plant m^-2^. The slope for the leaves of the upper half of the plant was not significant (*p* = 0.58). The R:FR ratio decreased significantly with PD both at mid-canopy height from 1.9 to 0.9 and at pot height below the canopy from 1.3 to 0.5. The estimated slopes were of similar magnitude with values of -0.038 for mid-canopy height and -0.027 at pot height (Fig 8).

In the D-trial, light interception over time was modeled by adjusting the logistic functions. The analysis of the curve parameters revealed a significant influence of PD on *L*_*max*_, *t*_*half*_, and *k*. Accordingly, the highest maximum light interception of 93% was obtained at the highest PD of 36 plants m^-2^, while it was only 74% at the lowest density of 12 plants m^-2^ (Fig 8). At this plant density, it took 2.71 weeks until half of the maximum light interception was reached, which corresponded approximately to the end of the vegetative phase (3 weeks). In comparison, the light interception at planting densities of 16, 24, and 36 plants m^-2^ at the same time point was 68%, 80%, and 87%. The pairwise comparisons of the curve parameters between the tested PD are shown in Table 4.

**Table 4:** Estimated mean values for light interception.

D-trial				V-trial	
Density [plants m^-2^]	Parameter				
	L_max_	t_half_	k	DVP [weeks]	light intercepted [%]
12	0.74 ± 0.03^c^	2.71 ± 0.14^a^	1.78 ± 0.28^a^	1	20.1 ± 1.4^d^
16	0.89 ± 0.01^b^	2.09 ± 0.09^b^	1.29 ± 0.1^a^	2	52.0 ± 1.4^c^
24	0.89 ± 0.01^b^	1.42 ± 0.21^c^	1.46 ± 0.21^a^	3	87.7 ± 1.4^b^
36	0.93 ± 0.01^a^	-0.14 ± 0.86^c^	0.67 ± 0.14^b^	4	93.1 ± 1.4^a^
*p-value*	0.005	0.007	0.03		< .0001

Results are presented as mean values ± estimated standard error (*n* = 3). Means within the same trial bearing at least one identical lowercase letter did not differ significantly at α = 0.05 within the same column.

In the V-trial, there was a significant lack-of-fit for the linear regression model. Hence, DVP was considered a qualitative variable. DVP showed significant effects on light interception. The maximum light interception reached a similarly high percentage (93.1 ± 1.4%) as in the highest PD in the D-trial for the plots of plants with a DVP of four weeks (Table 4). In comparison, the fraction of intercepted light decreased significantly by 5.8%, 44.1%, and 78.4% for plants with 3-week, 2-week, and 1-week DVP, respectively.

### 3.5 Area-based plant traits

**Fig 9:**
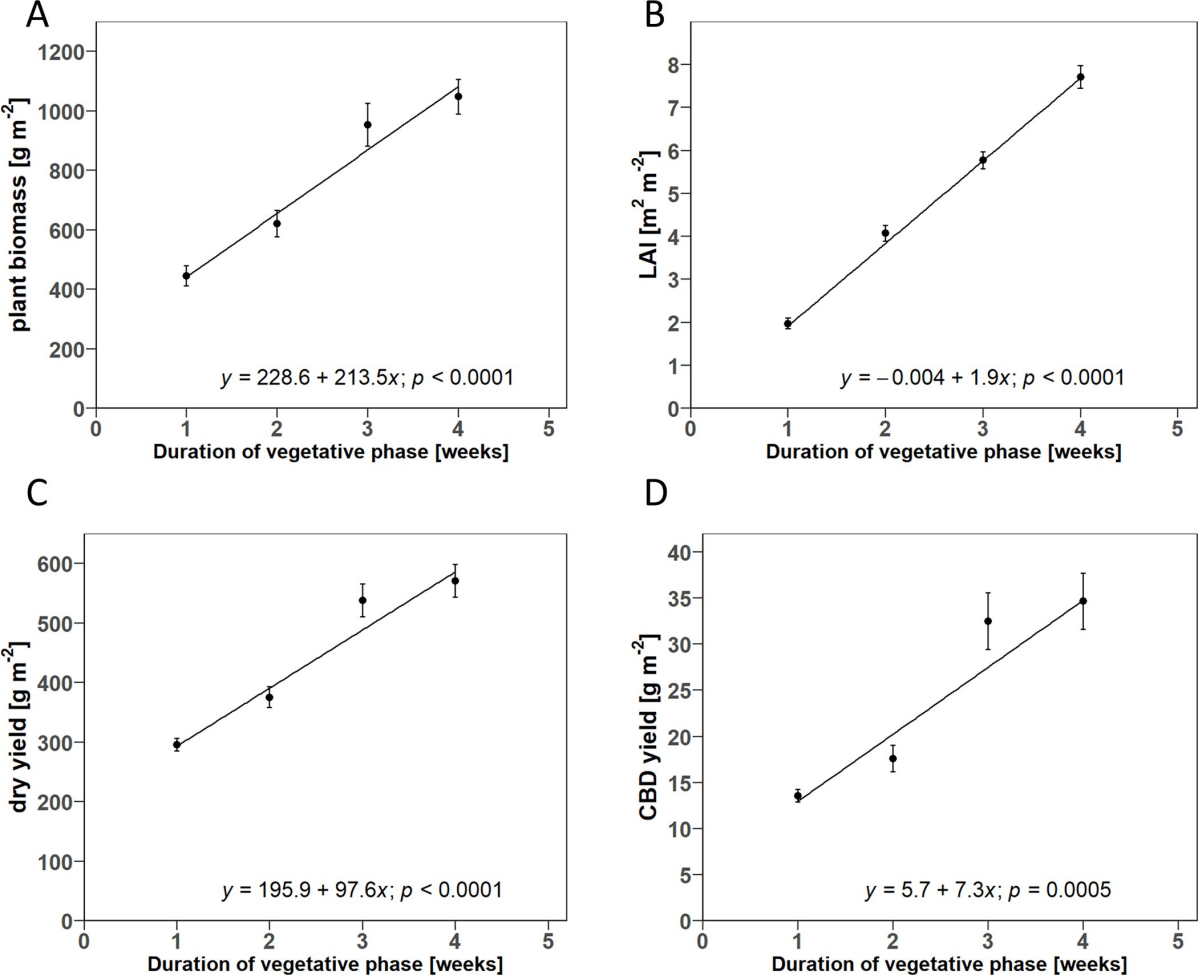
Scatter plots of the linear regression for the relationship between duration of the vegetative growth phase and A) total plant biomass per square meter at final harvest B) leaf area index (LAI) at third harvest, C) dry yield per square meter at final harvest, and D) CBD yield per square meter at final harvest. Point symbols show the estimated means for the respective plants, grown in the vegetative phase for 1,2,3, and 4 weeks. Error bars indicate the estimated standard error of the mean (*n* = 3). The solid line shows the fitted linear regression model. The *p*-values indicate whether the estimated slope terms of the final harvest are significantly different from zero.

A non-significant lack-of-fit test was found for LAI as well as total plant biomass and yield parameters on an area basis. Linear regression was accepted as a suitable model for these parameters in both D- and V-trials. In the V-trial, parameters on an area basis showed a positive correlation with DVP (Fig 9), following the results on a plant basis (Fig 5). The regression models showed an increase of 213.5 g m^-2^ week^-1^ for plant biomass, 1.9 m^2^ m^-2^ week^-1^ for LAI, 97.6 g m^-2^ week^- 1^ for dry inflorescence yield, and 7.3 g m^-2^ week^-1^ for CBD yield. The dry inflorescence yields achieved ranged from 295.36 g m^-2^ to 570.66 g m^-2,^ and the CBD yields ranged from 13.56 g m^-2^ to 34.66 g m^-2^.

**Fig 10:**
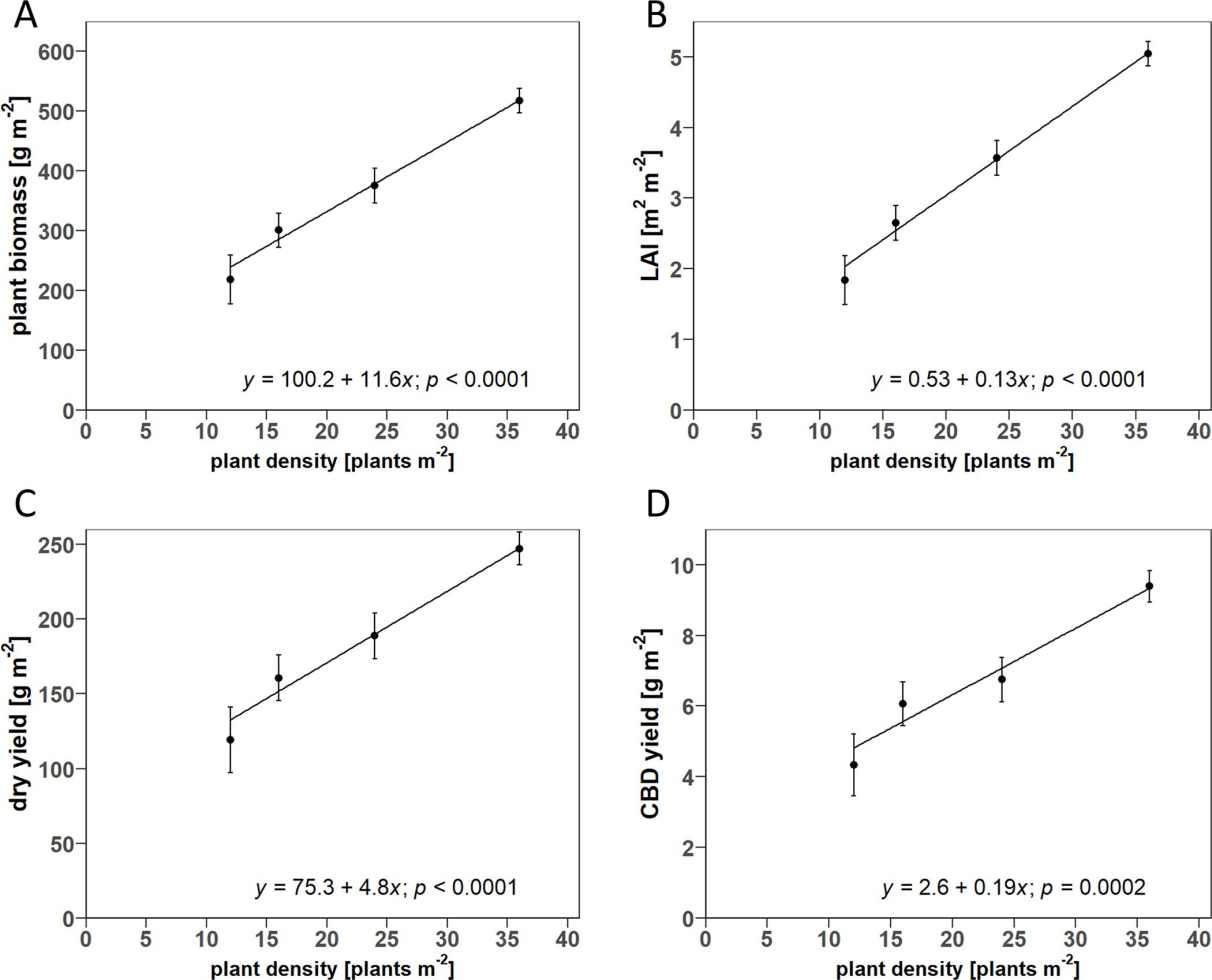
Scatter plots of the linear regression for the relationship between plant density and A) total plant biomass per square meter B) leaf area index (LAI), C) dry yield per square meter, and D) CBD yield per square meter. Point symbols show the estimated means for the respective plants, grown under densities of 12, 16, 24, and 36 plants m^-2^. Error bars indicate the estimated standard error of the mean (*n* = 3). The solid line shows the fitted linear regression model. *p*-values result from the global F-test for the fixed effect of plant density.

In the D-trial, the correlation of the area-related parameters with PD was also positive, in contrast to the results on an individual plant basis. The estimated increase in plant biomass was 11.6 g per additional plant m^-2^, while the estimated mean values of the tested PD increased from 218 to 518 g m^-2^ from lowest to highest PD. LAI ranged from 1.8–5 m^2^ m^-2^ from lowest to highest PD, with an estimated slope of 0.13 m^2^ m^-2^ per additional plant m^-2^. The dry yield increased by 4.8 g m^-2^ per additional plant m^-2^ and the CBD yield by 0.19 g m^-2^ per additional plant m^-2^ (Fig 10). The dry yields (119.2–247.08 g m^-2^) and CBD yields (4.34–9.39 g m^-2^) achieved in the D-trial were substantially lower than in the V-trial.

## 4. Discussion

To our knowledge, this is the first study to use regression analysis to analyze yield parameters of medicinal cannabis over a wide range of PDs. Similarly, we provide the first empirical evidence to derive correlations between DVP and final yield data.

Our results show that the biomass production per plant of all plant organs decreased linearly with increasing PD (Fig 4), which was compensated by an increasing number of plants per square meter. Consequently, LAI, total plant biomass, inflorescence, and cannabinoid yields per m^2^ linearly increased with PD (Fig 10). In contrast to the hypothesis, no saturation of area yields of cannabinoids and inflorescences was observed in response to increased PD within the tested ranges. The reduction in the biomass of individual plants in response to increasing PD could be attributed to the decrease of biomass in the lower canopy layers. Conversely, this meant a shift in biomass allocation to upper canopy layers, especially for inflorescences, with increased PD. The extension of DVP was generally associated with increased plant size and related growth parameters (Table 1). Consequently, a positive linear correlation was found for the inflorescence and cannabinoid yield on a single plant and area basis with DVP, following the hypothesis. In contrast, DVP had only a marginal effect on the biomass partitioning of plant organs between the upper and lower plant half (Fig 5). In general, the harvest index decreased both with increasing PD and DVP in favor of the stem fraction (Fig 6). Contrary to expectations, no significant change in the intra-plant cannabinoid concentration gradient from plant top to bottom was observed for PD or DVP. However, the CBD concentrations of plants with longer DVP (> 2 weeks) were generally increased, regardless of the inflorescence position within the plant (Table 3). Likewise, the size of the MAI was positively associated with DVP, while PD did not show significant effects on cannabinoid concentration or inflorescence size of different positions (Table 2). We interpret these results mainly as an argument for high-density production that maximizes area yields and, at the same time, promotes the proportion of higher concentrated inflorescence fractions from the upper canopy layers in the total yield.

### 4.1 Effect of PD

Our study joins a great body of research that has found a steady increase in LAI and, consequently, total plant biomass per area in response to increasing PD as a result of the increased light interception [14]. The linear increase in LAI is mainly linked to the increasing number of plants, as the effect of PD on leaf area of individual plants was found to be non-significant (Table 1). Similarly, the positive correlation of PD with the area yield of dry inflorescences and CBD area yield continues the trend of prior studies that tested this relationship for lower densities [7, 20, 21]. However, the results of previous studies for medicinal cannabis are inconclusive in this context, as especially for the higher PD in indoor cultivation (12–20 m^-2^) no significant differences in inflorescence area yield were determined [25, 26]. Nevertheless, it is worth noting that these studies each compared only a narrow range of plant densities using pairwise mean comparisons (e.g. 12 and 16 plants m^-2^), in contrast to the regression analysis applied in our study.

In greenhouse cultivation, especially in pot cultures with modern fertigation systems, inter-plant competition for water and nutrients due to increased PD is of little importance compared to light interception. Consequently, the reduction in plant biomass of individual plants as a typical response to increasing PD [7, 14] can be attributed to the reduced light transmission in lower canopy layers. Additional gas exchange measurements confirm the reduced photosynthetic activity in combination with higher SLA of the leaves of the lower half of the canopy (Fig 8), which represents a typical shading-induced response [13]. As a result, it could be shown that the reduction in biomass is mainly due to the decrease in leaf and stem growth in the lower half of the canopy, while PD had only a marginal effect on vegetative biomass growth in the upper half. Similarly, the dry inflorescence and CBD yield of the lower half of the plant decreased with PD. Nevertheless, the lack of saturation of agronomic yield parameters with increasing PD shows that plant densities in the range of 24–36 plants m^-2^ have their justification in indoor cultivation systems of medicinal cannabis. Cultivation under high PD allows plants to be kept smaller and growth cycles to be shorter [6] while improving CBD yield. High PD is, therefore, an attractive tool for the cultivation of medicinal cannabis for extraction purposes.

However, there are several indicators to assume that a further increase in planting density far beyond the tested range will not lead to further increases in yield per m^2^:

i) if one estimates the further course of the area yield based on the empirical relationships found for the individual plant yield (Fig 4), one finds that the inflorescence yield per m^2^ reaches its maximum at a PD of 39 plants m^-2^ and the CBD yield per m^2^ at a PD of 42 plants m^-2^ (S4 Table). However, it is questionable to what extent such an extrapolation is reasonable, especially since it can be assumed that the strict linear relationship is no longer valid for higher PD (> 24 plants m^-2^) but should better be described using an asymptotic model.

ii) the increase in biomass per m^2^ was accompanied by an improvement in light interception (Fig 8), which was maximized at the highest PD of 36 plants m^-2^ (LAI ~ 5). According to Beer’s law of light attenuation, any further increase in LAI will only lead to marginal increases in light interception [14].

iii) the overall plant size achieved in the D-trial was at the lower end of the known range from previous studies of the same genotype under similar growing conditions [8, 28, 30]. The effect of PD is related to plant size, as larger plants with more leaf area reach the LAI for the saturation point of light interception at lower densities.

In addition, there are other factors to consider that make a general recommendation regarding PD difficult. For example, cannabis genotypes can differ significantly in their growth behavior [33]. It is easy to imagine that a more fiber-like genotype, with a low branching tendency, allows higher PD than a bushy genotype with a strong tendency for lateral shoot growth. Producers should further consider the changes in microclimatic conditions at high PD [10] and the associated increased risk of diseases. However, in the D-trial, no visual symptoms of abiotic or biotic stress factors were found in any of the PDs tested. Therefore, future studies should investigate these factors in higher PD ranges.

A large part of the cultivation of medicinal cannabis is aimed at the direct commercialization of the inflorescences for medical applications. In addition to maximizing yield, the homogeneity of the chemical composition between the inflorescences from different positions within the canopy is a top priority. The natural light gradient within the canopy from the upper to lower layer is considered to be the main reason for the parallel gradient of cannabinoid concentration [7–9]. This gradient was also evident in the D- and V-trial results. Potential antidotes to this gradient are suitable pruning methods that favor light penetration into lower canopy layers [4, 8, 12]. Sub-canopy illumination has also been described as an effective tool [34]. In a previous study, it was reported that higher PD enhanced the cannabinoid concentration gradient as the cannabinoid concentration decreased in lower layers [7]. Our regression analysis could not confirm this effect, although the estimated mean values of the lower inflorescences followed this trend (Table 2). Note that the overall CBD concentration in our study was relatively low, and the expected effect size was correspondingly small, which could explain the lack of statistical significance (a similar explanation can also be given for the average inflorescence mass of the lower canopy layers). However, the increase in the concentration gradient does not automatically indicate increased chemical variability in the whole canopy. Such a conclusion ignores the proportional composition of the total yield from the individual inflorescence fractions. In the D-trial, at a PD of 12 plants m^-2^, the proportion of low-concentration inflorescences of the lower canopy layer was 53.8%, while this proportion was reduced to 32.3% for the highest density. Hence, increasing PD increases total yield and the proportion of higher concentrated inflorescence fractions from upper canopy layers, where lighting conditions can be better controlled. The MAI, in particular, is the fraction with the highest cannabinoid concentration, whose position in the canopy can be best controlled at the same time. By increasing the number of plants with increasing PD, the number of inflorescences of this fraction can be increased relatively easily, without affecting its average mass (Table 2). In summary, we therefore argue that increasing PD contributes to an enhancement of spatial chemical uniformity in indoor cultivation systems, as also previously stated in other studies [6, 35].

Morphological changes of the plant in response to altered PD are mainly due to the shade avoidance reaction of the plant, which is primarily triggered by an altered R:FR ratio in the crop and is perceived via the phytochrome apparatus of the plant [15]. The reduction of the R:FR ratio in the lower canopy layers with increasing PD is evident in our results (Fig 8). The increase in the SLA of the lower leaves shows the decreasing leaf thickness and is a typical response to the altered R:FR ratio. Similarly, the increased plant height of the high PD can also be attributed to the increasing internode elongation and confirms the results of previous studies for cannabis [7, 16]. Although lateral shoot length was not significantly affected by PD, the biomass of the lower lateral shoots decreased with increasing PD, indicating decreasing shoot thickness. This is the result of reduced assimilate production and restriction of secondary growth and is in line with the results from fiber-type hemp [17, 18, 36]. In contrast, drastic effects of light competition, such as impaired node formation and even reduced height growth, as reported for fiber-hemp production [36, 37], are not to be expected for indoor cultivation of medicinal cannabis, which is in line with our results. Firstly, the relevant range of densities for fiber-type hemp (180–270 plants m^-2^), where these effects occurred, is significantly higher than a commonly used PD in the production of medicinal cannabis. Secondly, these effects only appear as a reaction to the severe light competition after canopy closure [36]. The usual DVP in the indoor cultivation of medicinal cannabis is recommended at 1–4 weeks, thus, the overlapping time window of vegetative growth after full canopy closure is comparably small.

### 4.2 Effect of DVP

The morphological effects found in response to increasing DVP are in line with expectations. Increased plant height, lateral shoot length, and leaf area can be attributed primarily to the increased number of nodes on the main stem and lateral shoots (Table 1). Node formation is temperature-driven and can be estimated plant-specifically by temperature sum intervals [38]. Accordingly, the linear correlations found with DVP were to be anticipated. The total number of inflorescences per plant is also linked to the total number of nodes, as inflorescences in principle represent high-order lateral phytomers in a highly condensed form [39]. Therefore, the increase in inflorescence yield with prolonged DVP is primarily due to the increased total number of inflorescences rather than changes in the average inflorescence mass.

The positive linear relationships described for the different biomass fractions of single plants at final harvest are plausible since an extension of the vegetative phase entails an extension of the linear growth phase [28]. However, for the inflorescence dry yield per plant, our results are in direct contrast to the results of the meta-analysis by [29], where the relationship with DVP was negative. One possible reason for this difference is the consideration of environmental conditions, which are very likely to differ between studies (light intensity, pot size, water, and fertilization strategy) and represent a potential source of error as confounding factors when comparing data sets from several studies. In contrast, these factors are largely eliminated in controlled experiments. The range of plant biomass and plant height achieved for all DVP treatments was at the upper end of the previously reported range for the same genotype under similar conditions [28, 30]. Similarly, the achieved inflorescence yields per m^2^ fall within the typical range achieved in industrial production [2, 27]. It can be assumed that no severe stress factors influenced the relationships observed. The positive linear relationships between inflorescence yield and CBD yield per m^2^ support the assumption that area yields can be increased via extended DVP as an alternative to PD. Here, too, the improved leaf cover due to higher LAI and the associated improved light interception (Table 4) is the underlying cause. However, producers should bear in mind that in the case of DVP, the increase comes at a cost. Extended DVP means longer vegetation cycles and larger plants. This makes management more difficult during the cultivation period and may require additional care and pruning measures [6]. In addition, larger plants with many inflorescences, especially on the lower lateral shoots, make harvesting more time-consuming. Thus, it seems more sensible to set the desired yield level via the PD, as long as the available number of plants does not become a limiting factor.

Our results show that the CBD concentration significantly increased in all fractions for the treatments with DVP of 3 and 4 weeks. These results are in agreement with the modeled correlations between DVP and cannabinoid concentration [29]. At the same time, they directly contradict the often postulated dilution effect, according to which cannabinoid concentration decreases with increasing biomass yield [8, 40]. However, the elevated CBD concentrations in our case cannot be unequivocally attributed to DVP. Due to the higher biomass formation with longer DVP, it can be assumed that more nutrients were taken up from the substrate by plants with longer DVP. It, therefore, cannot be ruled out that the different nutrient levels during generative growth influenced the generally higher CBD concentrations. The increased leaf senescence towards the end of the growth cycle in the treatments of the longer DVP slightly indicates that nutrient supply became a limiting factor, which was compensated by increased nutrient translocation within the plant. The influence of nutritional status on cannabinoid concentration has already been discussed previously [40–42]. Furthermore, the average mass of the MAI was positively correlated with DVP. Nevertheless, in contrast to the hypothesis, no influence on the intra-plant cannabinoid concentration gradient could be found for DVP. To summarise, our results show no significant impact of DVP on spatial chemical variability. However, unlike PD, we could not show an effect on the size of inflorescense fractions from the upper and lower canopy layers. Assuming that different inflorescence fractions should be marketed separately due to the shown chemical gradient, producers seem to be better advised to focus on a higher PD with a short DVP for direct marketing of inflorescences than vice versa. However, the discussed effects on CBD concentration and average mass of MAI indicate that moderate DVPs (3–4 weeks) are justified in this context.

### 4.3 Limitations and outlook

Despite the plausible correlations obtained by the regression analysis, the presented trials had some limitations leaving room for improvement in future research. The plant size in the D-trial was generally small, which may have attenuated any density effects. Hence, the amplitude of the effect size for larger growing plants remains to be investigated. Similarly, the D-trial took place under low-light conditions. Cannabis has a comparatively high light saturation point of ~1500 μmol m^-2^ s^-1^, which allows cultivation under high light intensities [43]. Under these conditions, it is to be expected that density effects are generally greater. Likewise, the impact of the light spectrum in conjunction with altered PD should be investigated, as the light spectrum affects plant architecture [44, 45] and light penetration into the canopy [46], raising expectations of interactive effects with PD.

Future studies on DVP should aim to confirm the relationships found so far with cannabinoid concentration and investigate the influence on the chemical composition. It is important to include other chemotypes as well as other groups of secondary metabolites, such as terpenes, for the comparison of different PD and DVP. Likewise, it would be of benefit to quantify the effect of PD and DVP on inflorescence morphology and trichome density in future trials. In our study, we analyzed the effects of PD and DVP separately in different trials. It seems advisable to combine these factors in further studies, taking into account different genotypes, to derive specific recommendations for the cultivation of medicinal cannabis.

## 5. Conclusion

In this study, we performed a regression analysis for the separate effects of PD and DVP on plant growth, morphology, and spatial chemical variability of indoor cultivated medicinal cannabis. The main findings include: i) a positive linear model could be fitted for both DVP and PD within the tested ranges to the area yield for inflorescences and CBD; ii) neither DVP nor PD showed significant effects on the intra-plant gradient of CBD concentration. However, increasing PD led to a shift in yield composition in favor of the more desirable, higher-concentrated, and more homogeneous inflorescence fractions of the upper canopy layers; iii) prolonged DVP increased CBD concentration of all inflorescence fractions, and the average mass of MAI.

We conclude that producers should define their production systems by their PD. High density systems seem preferable due to favoring the proportion of inflorescence fractions from higher canopy layers and are associated with smaller, easier manageable plants. DVP should be adapted according to the selected PD. For the production goal of direct marketing of inflorescences, a DVP of 3 weeks seems to be a good compromise between the length of the growth cycle, plant size, and positive effects on yield and CBD concentration.

Future studies should focus more on the effects of PD and DVP on the variability of relevant chemical constituents (e.g. cannabinoids, terpenes). For more specific recommendations, PD and DVP should be combined as experimental factors. In addition, other factors, such as pruning, light intensity, spectrum, and genotype, should also be considered.

## Supporting information

S1 Fig Design of the D-trial. (TIF)

S1 Table Composition and initial properties of the substrates. (DOCX)

S2 Table F-test tables and results of the lack-of-fit test of the linear regression analysis for measured plant traits in the D-trial. (DOCX)

S3 Table F-test tables and results of the lack-of-fit test of the linear regression analysis for measured plant traits in the V-trial. (DOCX)

S4 Table Estimation of inflorescence and CBD yield per m^2^ in dependence on plant density (PD) based on empirical models for single plant yield. (DOCX)

S1 Data Raw data D-trial. (XLSX)

S2 Data Raw data V-trial. (XLSX)
