# Supplementary figures and images for: The effects of plant density and duration of vegetative growth phase on agronomic traits of medicinal cannabis (Cannabis sativa L.): A regression analysis
Source: PLoS One. 2024 Dec 30;19(12):e0315951. doi: 10.1371/journal.pone.0315951 (PMC11684660; doi:10.1371/journal.pone.0315951)

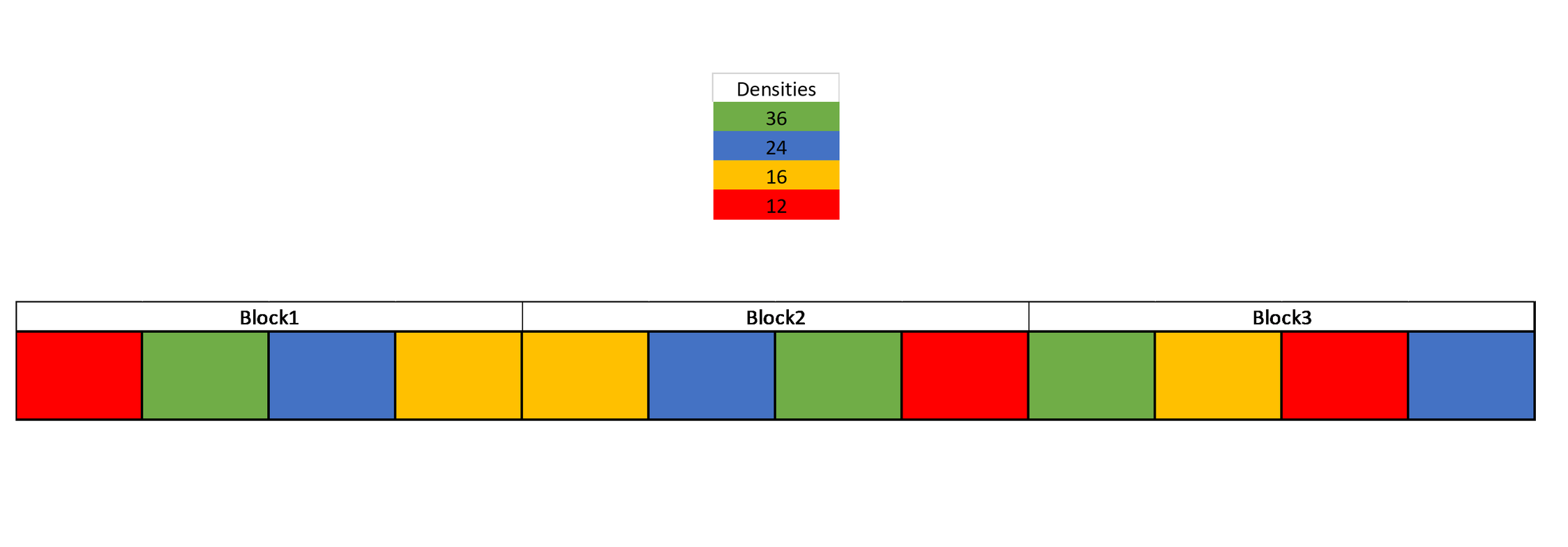

Supplement: S1 Fig — (TIF) [file pone.0315951.s001.tif]
