# Supplementary material for: The effects of plant density and duration of vegetative growth phase on agronomic traits of medicinal cannabis (Cannabis sativa L.): A regression analysis
Source: PLoS One. 2024 Dec 30;19(12):e0315951. doi: 10.1371/journal.pone.0315951 (PMC11684660; doi:10.1371/journal.pone.0315951)
Supplement: S1 Table — (DOCX) [file pone.0315951.s002.docx]

**Table S1. Composition and initial properties of the substrates**

|  | Peat | | Coco-coir | | |
| --- | --- | --- | --- | --- | --- |
| composition | 35% | milled-peat | 35% | coir fine removed |  |
|  | 30% | sod-cut peat fraction 2 | 30% | coir fiber |  |
|  | 15% | green fiber | 15% | green fiber |  |
|  | 20% | perlite | 20% | Perlite |  |
|  |  |  |  |  |  |
| pH (H_2_O) | 5.99 | | 6.03 | | |
| EC (dS m^-1^) | 0.246 | | 0.289 | | |
| bulk density (g cm^-3^) | 0.099 | | 0.096 | | |
