# Supplementary material for: The effects of plant density and duration of vegetative growth phase on agronomic traits of medicinal cannabis (Cannabis sativa L.): A regression analysis
Source: PLoS One. 2024 Dec 30;19(12):e0315951. doi: 10.1371/journal.pone.0315951 (PMC11684660; doi:10.1371/journal.pone.0315951)
Supplement: S2 Table — (DOCX) [file pone.0315951.s003.docx]

**Table S2. F-test tables and results of the lack-of-fit test of the linear regression analysis for measured plant traits in the D-trial**

| Fixed Effects | DF | Morphology (no position effects) | | | | | | |
| --- | --- | --- | --- | --- | --- | --- | --- | --- |
|  |  | Leaf area**^1^** | Height | Shoots | No. Nodes | No. Inflorescences**^1^** | |  |
| Block | 2 | 0.7056 | 0.3585 | 0.3585 | 0.9725 | 0.1005 |  | |
| Density[D] | 1 | 0.0678 | 0.0206 | 0.0206 | 0.1986 | 0.6255 |  | |
| Time [T] | 7 | - | <.0001 | <.0001 | <.0001**^2^** | - |  | |
| D $\times$ T | 7 | - | 0.0177 | 0.0709 | 0.7556**^2^** | - |  | |
| lack-of-fit | | 0.5955 | 0.6075 | 0.7391 | 0.3263 | 0.0615 |  | |
| Fixed Effects | DF | Single plant parameters (different positions) | | | | | | |
|  |  | Leaf | Stem | Yield | CBD yield | CBD conc. (%) | Avg. Infl. Mass | |
| Block | 2 | 0.7279 | 0.4081 | 0.9145 | 0.8354 | 0.6739 | 0.2565 | |
| Density [D] | 1 | 0.0757 | 0.7277 | 0.0202 | 0.0038 | 0.4438 | 0.1662 | |
| Position [P] | 1 | <.0001 | <.0001 | 0.0332 | 0.4292 | <.0001 | <.0001 | |
| D $\times$ P | 1 | 0.0006 | 0.0031 | 0.0007 | 0.0197 | 0.2092 | 0.7109 | |
| lack-of-fit | | 0.4063 | 0.5766 | 0.7031 | 0.5345 | 0.3085 | 0.7538 | |
| Fixed Effects | DF | Organ fractions (no position effects) | | | | | | |
|  |  | Inflorescences | | Leaf | | Stem | | |
| Block | 2 | 0.5977 | | 0.8379 | | 0.1362 | | |
| Density | 1 | 0.001 | | 0.1468 | | 0.0003 | | |
| lack-of-fit | | 0.7788 | | 0.2477 | | 0.5615 | | |
| Fixed Effects | DF | Additional Parameters (different positions) | | | | | | |
|  |  | A_max_ | | SLA | | R:FR | | |
| Block | 2 | 0.534 | | 0.9585 | | 0.3589 | | |
| Density [D] | 1 | 0.0192 | | 0.0572 | | 0.0028 | | |
| Position [P] | 1 | 0.2626 | | <.0001 | | 0.0002 | | |
| D $\times$ P | 1 | 0.0493 | | 0.0005 | | 0.0004 | | |
| lack-of-fit | | 0.9004 | | 0.9427 | | 0.4364 | | |
| Fixed Effects | DF | Area-based parameters (no position effects) | | | | | | |
|  |  |  | Tot. biomass | LAI | Yield | CBD yield |  | |
| Block | 2 |  | 0.4779 | 0.3174 | 0.5737 | 0.4681 |  | |
| Density | 1 |  | <.0001 | <.0001 | 0.0001 | 0.0002 |  | |
| lack-of-fit | |  | 0.77 | 0.7735 | 0.7116 | 0.5722 |  | |

^1^ parameters were only measured once at final harvest (no effect for time)

^2^ DF = 6
