# Supplementary material for: The effects of plant density and duration of vegetative growth phase on agronomic traits of medicinal cannabis (Cannabis sativa L.): A regression analysis
Source: PLoS One. 2024 Dec 30;19(12):e0315951. doi: 10.1371/journal.pone.0315951 (PMC11684660; doi:10.1371/journal.pone.0315951)
Supplement: S3 Table — (DOCX) [file pone.0315951.s004.docx]

**Table S3. F-test tables and results of the lack-of-fit test of the linear regression analysis for measured plant traits in the V-trial**

| Fixed Effects | DF | Morphology (no position effects) | | | | | | |
| --- | --- | --- | --- | --- | --- | --- | --- | --- |
|  |  | Leaf area | Height | Shoots | No. Nodes | No. Inflorescences**^2^** | |  |
| Block | 2 | 0.463 | <.0001 | 0.7246 | 0.6226 | 0.8083 |  | |
| DVP [D] | 1 | <.0001 | <.0001 | <.0001 | <.0001 | <.0001 |  | |
| Substrate [S] | 1 | 0.482 | 0.0058 | 0.1478 | 0.253 | 0.1274 |  | |
| Time [T] | 3 | 0.0251**^1^** | <.0001 | <.0001 | <.0001 | - |  | |
| D $\times$ S | 1 | 0.6798 | 0.0012 | 0.0498 | 0.4539 | 0.0765 |  | |
| D $\times$ T | 3 | 0.0567**^1^** | <.0001 | 0.5859 | 0.0119 | - |  | |
| S $\times$ T | 3 | 0.9413**^1^** | 0.7439 | 0.8461 | 0.7714 | - |  | |
| D $\times$ S $\times$ T | 3 | 0.4388**^1^** | 0.821 | 0.8247 | 0.7831 | - |  | |
| lack-of-fit | | 0.0573 | 0.1537 | 0.2884 | 0.175 | 0.2949 |  | |
| Fixed Effects | DF | Single plant parameters (different positions) | | | | | | |
|  |  | Leaf | Stem | Yield | CBD yield | CBD conc. (%)**^4^** | Avg. Infl. Mass | |
| Block | 2 | 0.2015 | 0.1754 | 0.1945 | 0.2652 | 0.5219 | 0.3621 | |
| DVP [D] | 1 | 0.0003 | <.0001 | 0.0001 | 0.0011 | <.0001 | 0.021 | |
| Position [P] | 1 | 0.6854 | 0.4408 | 0.6022 | 0.7534 | 0.0012 | 0.0044 | |
| Substrate [S] | 1 | 0.4761 | 0.259 | 0.7626 | 0.7487 | 0.1427 | 0.956 | |
| D $\times$ S | 1 | 0.2579 | 0.3116 | 0.4166 | 0.4136 | 0.4264 | 0.5242 | |
| D $\times$ P | 1 | 0.0429 | <.0001 | 0.7322 | 0.3584 | 0.7636 | 0.0249 | |
| P $\times$ S | 1 | 0.3045 | 0.0279 | 0.8939 | 0.5038 | 0.9631 | 0.7831 | |
| D $\times$ P $\times$ S | 1 | 0.1759 | 0.0644 | 0.5753 | 0.2387 | 0.077 | 0.7007 | |
| lack-of-fit | | 0.6311 | 0.3499 | 0.1085 | 0.1019 | 0.0063 | 0.7777 | |
| Fixed Effects | DF | Organ fractions (no position effects) | | | | | | |
|  |  | Inflorescences | | Leaf | | Stem | | |
| Block | 2 | 0.683 | | 0.0736 | | 0.5881 | | |
| DVP [D] | 1 | <.0001 | | 0.0292 | | <.0001 | | |
| Time [T] | 4 | <.0001**^3^** | | <.0001 | | <.0001 | | |
| Substrate [S] | 1 | 0.0035 | | 0.6338 | | <.0001 | | |
| S $\times$ T | 4 | 0.08**^3^** | | 0.0174 | | 0.2765 | | |
| D $\times$ S | 1 | 0.0166 | | 0.3048 | | 0.0501 | | |
| D $\times$ T | 4 | 0.2346**^3^** | | <.0001 | | 0.0013 | | |
| D $\times$ S $\times$ T | 4 | 0.1073**^3^** | | 0.2924 | | 0.5353 | | |
| lack-of-fit | | 0.7934 | | 0.9873 | | 0.3882 | | |
| Fixed Effects | DF | Area-based parameters (no position effects) | | | | | | |
|  |  |  | Tot. biomass | LAI | Yield | CBD yield**^2^** |  | |
| Block | 2 |  | 0.3635 | 0.463 | 0.0157 | 0.721 |  | |
| DVP [D] | 1 |  | 0.0005 | <.0001 | <.0001 | 0.0005 |  | |
| Time [T] | 4 |  | <.0001 | 0.482**^1^** | <.0001**^3^** | - |  | |
| Substrate [S] | 1 |  | 0.1257 | 0.0251 | 0.4999 | 0.271 |  | |
| S $\times$ T | 4 |  | 0.8556 | 0.6798**^1^** | 0.9311**^3^** | - |  | |
| D $\times$ S | 1 |  | 0.6071 | 0.0567 | 0.694 | 0.1082 |  | |
| D $\times$ T | 4 |  | <.0001 | 0.9413**^1^** | 0.0004**^3^** | - |  | |
| D $\times$ S $\times$ T | 4 |  | 0.4148 | 0.4388**^1^** | 0.2355**^3^** | - |  | |
| lack-of-fit | |  | 0.0553 | 0.0573 | 0.2211 | 0.0827 |  | |

^1^ DF = 1 (only measured for second and third harvest)

^2^ parameters were only measured at final harvest (no effect for time)

^3^ DF = 2 (no yield at first two harvests)

^4^ DVP was fitted as qualitative, as lack-of-fit was significant
