# Supplementary material for: The effects of plant density and duration of vegetative growth phase on agronomic traits of medicinal cannabis (Cannabis sativa L.): A regression analysis
Source: PLoS One. 2024 Dec 30;19(12):e0315951. doi: 10.1371/journal.pone.0315951 (PMC11684660; doi:10.1371/journal.pone.0315951)
Supplement: S4 Table — (DOCX) [file pone.0315951.s005.docx]

**Table S4. Estimation of inflorescence and CBD yield per m^2^ in dependence on plant density (PD) based on empirical models for single plant yield**

| PD | Infl yield upper half [g] | Infl yield lower half [g] | CBD yield upper half [g] | CBD yield lower [g] | Infl yield plant [g] | CBD yield plant [g] | Infl yield  [g m^-2^] | CBD yield  [g m^-2^] |
| --- | --- | --- | --- | --- | --- | --- | --- | --- |
| 12 | 5.16 | 5.32 | 0.23 | 0.15 | 10.48 | 0.38 | 125.76 | 4.55 |
| 13 | 5.14 | 5.18 | 0.22 | 0.15 | 10.32 | 0.37 | 134.16 | 4.86 |
| 14 | 5.12 | 5.04 | 0.22 | 0.14 | 10.16 | 0.37 | 142.24 | 5.16 |
| 15 | 5.1 | 4.9 | 0.22 | 0.14 | 10 | 0.36 | 150 | 5.45 |
| 16 | 5.08 | 4.76 | 0.22 | 0.14 | 9.84 | 0.36 | 157.44 | 5.73 |
| 17 | 5.06 | 4.62 | 0.22 | 0.13 | 9.68 | 0.35 | 164.56 | 6.00 |
| 18 | 5.04 | 4.48 | 0.22 | 0.13 | 9.52 | 0.35 | 171.36 | 6.25 |
| 19 | 5.02 | 4.34 | 0.22 | 0.12 | 9.36 | 0.34 | 177.84 | 6.50 |
| 20 | 5 | 4.2 | 0.22 | 0.12 | 9.2 | 0.34 | 184 | 6.74 |
| 21 | 4.98 | 4.06 | 0.22 | 0.11 | 9.04 | 0.33 | 189.84 | 6.97 |
| 22 | 4.96 | 3.92 | 0.22 | 0.11 | 8.88 | 0.33 | 195.36 | 7.18 |
| 23 | 4.94 | 3.78 | 0.22 | 0.11 | 8.72 | 0.32 | 200.56 | 7.39 |
| 24 | 4.92 | 3.64 | 0.21 | 0.10 | 8.56 | 0.32 | 205.44 | 7.59 |
| 25 | 4.9 | 3.5 | 0.21 | 0.10 | 8.4 | 0.31 | 210 | 7.77 |
| 26 | 4.88 | 3.36 | 0.21 | 0.09 | 8.24 | 0.31 | 214.24 | 7.95 |
| 27 | 4.86 | 3.22 | 0.21 | 0.09 | 8.08 | 0.30 | 218.16 | 8.11 |
| 28 | 4.84 | 3.08 | 0.21 | 0.08 | 7.92 | 0.30 | 221.76 | 8.27 |
| 29 | 4.82 | 2.94 | 0.21 | 0.08 | 7.76 | 0.29 | 225.04 | 8.41 |
| 30 | 4.8 | 2.8 | 0.21 | 0.08 | 7.6 | 0.28 | 228 | 8.54 |
| 31 | 4.78 | 2.66 | 0.21 | 0.07 | 7.44 | 0.28 | 230.64 | 8.67 |
| 32 | 4.76 | 2.52 | 0.21 | 0.07 | 7.28 | 0.27 | 232.96 | 8.78 |
| 33 | 4.74 | 2.38 | 0.21 | 0.06 | 7.12 | 0.27 | 234.96 | 8.88 |
| 34 | 4.72 | 2.24 | 0.21 | 0.06 | 6.96 | 0.26 | 236.64 | 8.97 |
| 35 | 4.7 | 2.1 | 0.21 | 0.05 | 6.8 | 0.26 | 238 | 9.06 |
| 36 | 4.68 | 1.96 | 0.20 | 0.05 | 6.64 | 0.25 | 239.04 | 9.13 |
| 37 | 4.66 | 1.82 | 0.20 | 0.04 | 6.48 | 0.25 | 239.76 | 9.19 |
| 38 | 4.64 | 1.68 | 0.20 | 0.04 | 6.32 | 0.24 | 240.16 | 9.24 |
| 39 | 4.62 | 1.54 | 0.20 | 0.04 | 6.16 | 0.24 | **240.24** | 9.28 |
| 40 | 4.6 | 1.4 | 0.20 | 0.03 | 6 | 0.23 | 240 | 9.31 |
| 41 | 4.58 | 1.26 | 0.20 | 0.03 | 5.84 | 0.23 | 239.44 | 9.32 |
| 42 | 4.56 | 1.12 | 0.20 | 0.02 | 5.68 | 0.22 | 238.56 | **9.33** |
| 43 | 4.54 | 0.98 | 0.20 | 0.02 | 5.52 | 0.22 | 237.36 | 9.33 |
| 44 | 4.52 | 0.84 | 0.20 | 0.01 | 5.36 | 0.21 | 235.84 | 9.32 |
| 45 | 4.5 | 0.7 | 0.20 | 0.01 | 5.2 | 0.21 | 234 | 9.29 |
| 46 | 4.48 | 0.56 | 0.20 | 0.01 | 5.04 | 0.20 | 231.84 | 9.26 |
| 47 | 4.46 | 0.42 | 0.19 | 0.00 | 4.88 | 0.20 | 229.36 | 9.22 |
| 48 | 4.44 | 0.28 | 0.19 | 0.00 | 4.72 | 0.19 | 226.56 | 9.16 |
| 49 | 4.42 | 0.14 | 0.19 |  | 4.56 | 0.19 | 223.44 | 9.10 |
| 50 | 4.4 | 0 | 0.19 |  | 4.4 | 0.18 | 220 | 9.02 |
